# Generation of Tetracycline and Rifamycin Resistant *Chlamydia Suis* Recombinants

**DOI:** 10.3389/fmicb.2021.630293

**Published:** 2021-06-30

**Authors:** Hanna Marti, Sankhya Bommana, Timothy D. Read, Theresa Pesch, Barbara Prähauser, Deborah Dean, Nicole Borel

**Affiliations:** ^1^Vetsuisse Faculty, Institute of Veterinary Pathology, University of Zurich, Zurich, Switzerland; ^2^Division of Infectious Diseases, Departments of Medicine and Pediatrics, University of California San Francisco School of Medicine, San Francisco, CA, United States; ^3^Division of Infectious Diseases, Department of Medicine, Emory University School of Medicine, Atlanta, GA, United States; ^4^Department of Human Genetics, Emory University School of Medicine, Atlanta, GA, United States; ^5^Joint Graduate Program in Bioengineering, University of California, San Francisco, San Francisco, CA, United States; ^6^Joint Graduate Program in Bioengineering, University of California, Berkeley, Berkeley, CA, United States

**Keywords:** co-culture, co-infection, recombination, rifampin resistance, RNAP, *rpoB*, Tet-island, *Chlamydiaceae*

## Abstract

The *Chlamydiaceae* are a family of obligate intracellular, gram-negative bacteria known to readily exchange DNA by homologous recombination upon co-culture *in vitro*, allowing the transfer of antibiotic resistance residing on the chlamydial chromosome. Among all the obligate intracellular bacteria, only *Chlamydia* (*C*.) *suis* naturally integrated a tetracycline resistance gene into its chromosome. Therefore, in order to further investigate the readiness of *Chlamydia* to exchange DNA and especially antibiotic resistance, *C. suis* is an excellent model to advance existing co-culture protocols allowing the identification of factors crucial to promote homologous recombination *in vitro*. With this strategy, we co-cultured tetracycline-resistant with rifamycin group-resistant *C. suis*, which resulted in an allover recombination efficiency of 28%. We found that simultaneous selection is crucial to increase the number of recombinants, that sub-inhibitory concentrations of tetracycline inhibit rather than promote the selection of double-resistant recombinants, and identified a recombination-deficient *C. suis* field isolate, strain SWA-110 (1-28b). While tetracycline resistance was detected in field isolates, rifampicin/rifamycin resistance (RifR) had to be induced *in vitro*. Here, we describe the protocol with which RifR *C. suis* strains were generated and confirmed. Subsequent whole-genome sequencing then revealed that G530E and D461A mutations in *rpoB*, a gene encoding for the β-subunit of the bacterial RNA polymerase (RNAP), was likely responsible for rifampicin and rifamycin resistance, respectively. Finally, whole-genome sequencing of recombinants obtained by co-culture revealed that recombinants picked from the same plate may be sibling clones and confirmed *C. suis* genome plasticity by revealing variable, apparently non-specific areas of recombination.

## Introduction

The obligate intracellular bacterial family *Chlamydiaceae* comprises a single genus with a wide range of species that infect human and animal hosts (Sachse and Borel, 2020). The most common cause of bacterial sexually transmitted infections (STI) and infectious blindness worldwide is *Chlamydia* (*C*.) *trachomatis* (Jordan et al., 2020). It is also the most widely-studied *Chlamydia* species. The closest phylogenetic relatives to *C. trachomatis* are the murine species *C. muridarum* and the porcine species *C. suis*. The latter is commonly detected in domesticated pigs and wild boar (Hotzel et al., 2004; Schautteet and Vanrompay, 2011; Sachse and Borel, 2020).

While murine genital infections with *C. muridarum* are often used as a model to study genital infections in humans, the natural prevalence and pathogenicity of this organism in mice remains unknown (Ramsey et al., 2016; Sachse and Borel, 2020, p. 406). In contrast, *C. suis* is highly prevalent in the gastrointestinal tract of pigs (Hoffmann et al., 2015) and is associated with ocular and genital tract disease (Schautteet and Vanrompay, 2011; Chahota et al., 2018). Moreover, *C. suis* is a zoonotic pathogen, causing asymptomatic or very mild ocular infection among pig farm workers as well as pharyngeal and rectal infections among abattoir employees (De Puysseleyr et al., 2014, 2017).

Although the clinical impact of *C. suis* on the pig industry and human health appears to be minor, this species has gained a lot of attention since the discovery that they can carry tetracycline resistance in 1998 (Lenart et al., 2001; Schautteet and Vanrompay, 2011). Specifically, *C. suis* is the only known species of the *Chlamydiales* order or other obligate intracellular bacteria (Vanrompay et al., 2018) that has naturally acquired a tetracycline resistance-conferring efflux pump encoded by the tetracycline resistance gene *tetA*(C) and its repressor *tetR*(C) (Dugan et al., 2004). This discovery is highly significant because *C. suis* has been found to co-infect—along with *C. trachomatis—*the eyes of trachoma patients in Nepal and Sudan, indicating that natural co-infection of the two species can occur in humans (Dean et al., 2013; Ghasemian et al., 2018).

There have been multiple reports about the presence of tetracycline-resistant (TetR) strains in pigs worldwide, including China (Li et al., 2017), Israel, Cyprus, and Western European countries such as Switzerland, Austria, Germany, Italy, and Belgium (Di Francesco et al., 2008; Borel et al., 2012; Schautteet et al., 2013; Wanninger et al., 2016; Peisker et al., 2018; Unterweger et al., 2020), as well as the USA (Dugan et al., 2004). However, to date, none of the zoonotically transmitted *C. suis* infections screened for tetracycline resistance (De Puysseleyr et al., 2014, 2017; Kieckens et al., 2018) have tested positive. Nonetheless, the potential for *C. trachomatis* to acquire TetR from *C. suis* should be closely monitored, especially since *in vitro* co-culture experiments with *C. suis* and *C. trachomatis* have shown successful uptake of *tetA*(C)/*tetR*(C) and neighboring *C. suis* genes by *C. trachomatis* (Suchland et al., 2009).

In TetR *C. suis, tetA*(C) and *tetR*(C) are part of a 6–14.5 kilobase pair (kbp) long genomic Tet-island on the chromosome, consistently inserting into the non-functional invasin (*inv*) gene located between two ribosomal RNA (rrn) operons (Dugan et al., 2004; Burall et al., 2007; Joseph et al., 2016; Marti et al., 2017; Seth-Smith et al., 2017). Interestingly, it is still not entirely clear how the Tet-island spreads among *C. suis* strains, although several studies have contributed to our current state of knowledge. For example, whole-genome analysis has shown that, while *C. suis* strains have likely gained and lost the Tet-island several times in the history of the species (Seth-Smith et al., 2017), the consistent site of insertion indicates that the original acquisition of the Tet-island was a unique event and resulted from the integration of a resistance-conferring plasmid originating from the bacterial class of Betaprotebacteria (Joseph et al., 2016). Additionally, genomic studies have also shown that the Tet-island is likely a recent addition to the *C. suis* chromosome (Joseph et al., 2016; Seth-Smith et al., 2017) possibly coinciding with the discovery and use of tetracycline in the pig industry (Joseph et al., 2016; Sachse and Borel, 2020, p. 414–415). These findings were further supported by the clear indication that selective pressure promoted the occurrence of TetR *C. suis* strains (Borel et al., 2012). Finally, a small-scale *in vitro* study demonstrated that TetS *C. suis* strains can obtain the Tet-island *via* recombination solely in the presence of tetracycline—without a counter-selectable marker—indicating the ease with which these events can occur (Marti et al., 2017).

The aim of the present study was to build on these previous findings and to establish an *in vitro* co-culture protocol that allows detection of *C. suis* recombinants in possession of a Tet-island while retaining the genomic background of the TetS recipient strain, and to identify factors that influence recombination efficiency. Here, we discuss the nuances of the methodology and the effect of different conditions and antibiotic selection on efficiency. In addition, we establish a protocol to generate and analyze rifamycin group resistant *C. suis* strains.

## Materials and Methods

### Antibiotics, Cells, and Culture Media

Reagents used in this study included rifampicin (Merck, Darmstadt, Germany; Sigma-Aldrich, St. Louis, Missouri, USA, Cat. No. R3501-250MG; 10 mg/ml in DMSO, filtered through 0.2 μm filters), rifamycin (Merck; Sigma-Aldrich Cat. No. R8626-1G; 10 mg/ml in 95% EtOH, filtered through 0.22 μm filter), and tetracycline (Merck; Sigma-Aldrich Cat. No. T7660-25G; 10 mg/ml in ddH_2_O, filtered through 0.22 μm filters).

All cell culture experiments were performed in LLC-MK2 cells (continuous Rhesus monkey kidney cell line, kindly provided by IZSLER Brescia, Italy), which were grown at 37°C and 5% CO_2_. Growth medium was used for cell seeding, propagation of *C. suis* and maintenance whereas infection medium was used for all cell culture experiments. LLC-MK2 growth medium consisted of 500 ml Minimal Essential Medium (MEM) with Earle's salts, 25 mM HEPES, without L-Glutamine (GIBCO, Invitrogen, Carlsbad, CA, USA) supplemented with 10% FCS and 2 mM GlutaMAX-I (200 mM, GIBCO), and 0.4 g D-(+)-glucose (Sigma-Aldrich). Infection medium consisted of 500 ml MEM supplemented with 20% FCS, 2 mM GlutaMAX-I (200 mM, GIBCO), 2 g D-(+)-glucose (Sigma-Aldrich), with or without 1.5 μg/ml cycloheximide (Sigma-Aldrich) as described (Wanninger et al., 2016), with minor changes. Sucrose phosphate glutamate (SPG) buffer consisted of 218 mM sucrose (Sigma-Aldrich, St. Louis, MO, USA), 3.76 mM KH_2_PO_4_ (Sigma-Aldrich), 7.1 mM K_2_HPO_4_ (Merck Eurolab AG, Dietlikon, Switzerland), and 5 mM GlutaMAX-100 (GIBCO) (Leonard et al., 2017).

### *Chlamydia* Strains

Table 1 lists the details of the *C. suis* strains used in this study, including their susceptibility to tetracycline (TetR or TetS). For experimental infections, SPG stocks containing semi-purified elementary bodies (EB) of each strain were prepared as described previously (Leonard et al., 2017), with modifications. Briefly, stocks were passaged in LLC-MK2 cells for up to five passages until six or eight T75 flasks (TPP, Trasadingen, Schweiz) were infected at a rate of 75–100%. Crude stocks were produced with the help of mechanical disruption by scraping the infected cells into culture medium and vortexing the suspension at maximum speed for 1 min with sterile 5 mm ∅ glass beads (Sigma-Aldrich). Cellular debris was removed by centrifugation (500 g, 4°C, 10 min). Chlamydiae were then pelleted (10,000 g, 4°C, 45 min) and resuspended in 3–6 ml of SPG medium depending on the size of the pellet. Stocks were aliquoted, stored at −80°C, and the concentration of each stock was determined using titration by sub-passage (Supplementary Data 1).

**Table 1:** List of *C. suis* strains used in the *in vitro* co-culture study.

**Strain (alt. name)**	**References**	**Resistance^a^**	**RifS: Accession No. and MIC^b^**	**RifR: Accession No. and MIC^c^**	***rpoB* mutation**
Type strain S45 (S45/6)	Kaltenboeck and Storz, 1992; Joseph et al., 2016	TetS, RifR (rifampicin)	SRS1519282, 0.002 μg/ml	CP063064, 0.015 μg/ml	G530E
SWA-94 (10-26b)	Wanninger et al., 2016	TetS, RifR (rifamycin)	PRJEB17986, 0.5–1 μg/ml	CP063063, 4–8 μg/ml	D461A
SWA-107 (5-27b)	Wanninger et al., 2016	TetR, RifS	PRJEB17986	–	–
SWA-110 (1-28b)	Wanninger et al., 2016	TetR, RifS	PRJEB17986	–	–
SWA-111 (1-28a)	Wanninger et al., 2016	TetS, RifR (rifamycin)	PRJEB17986, 0.5–1 μg/ml	CP063062, 8–16 μg/ml	D461A
SWA-141 (4-29b)	Wanninger et al., 2016	TetR, RifS	PRJEB17986	–	–

a *Tet, tetracycline; Rif, rifamycin/rifampicin; S, sensitive; R, resistant. Rif resistance was induced as described in the Methods*.

b, c *NCBI accession number, minimal inhibitory concentration (MIC, in μg/ml) of the RifS and RifR strains to rifampicin/rifamycin*.

### Generation of Recombinants and Analysis: A Brief Overview

**Figure 1:**
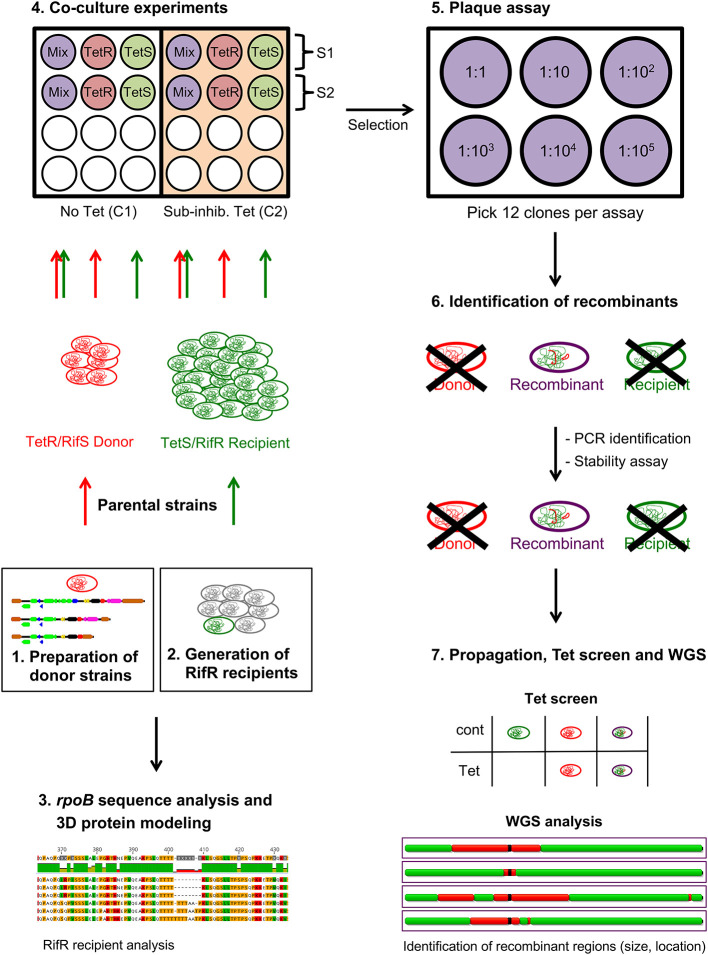
Overview of the study workflow. (1) TetR donor strains (depicted in red) were prepared and analyzed concerning their Tet-island. In parallel, (2) rifamycin resistant (RifR) TetS recipient strains (depicted in green) were generated. (3) Following single nucleotide polymorphism (SNP) analysis, the *rpoB* gene of the recipient strains was compared against their original strain, followed by 3D protein modeling with the Geneious Prime (version 2019.2.3, Biomatters, Ltd., Auckland, New Zealand) and the PyMOL software (The PyMol Molecular Graphics System, Version 1.2r3pre, Schrödinger, LLC., New York, NY, USA). Next, (4) these parental strains were cultured either alone or together (Mix, purple) in a 24-well plate in the presence (orange, Condition C2) or absence (white, C1) of subinhibitory concentrations of tetracycline. Following selection in inhibitory concentrations of tetracycline and rifampicin/rifamycin immediately after co-culture (Selection S1) or after a selection-free passage (S2), (5) co-cultures were subjected to plaque assays, each performed in a 6-well plate, with twelve picks per assay. (6) Putative recombinants were then identified by conventional PCRs [presence of the *tetA*(C) gene and TetS recipient-specific sequences; absence of TetR donor-specific regions] and further processed employing another round of selection, stability assays and re-confirmation by PCR. Next, (7) stocks of a limited number of suspected recombinants were prepared, screened for tetracycline resistance (Tet screen) and whole–genome sequenced (WGS) using the Illumina MiSeq platform. Recombinant regions were then identified and characterized regarding their size and location using Harvest software tool Gingr (https://harvest.readthedocs.io/en/latest/) and Geneious Prime.

In order to generate tetracycline and rifamycin resistant (TetR/RifR) recombinants that carry the Tet-island of a TetR/RifS donor strain while retaining the genomic background of a TetS/RifR recipient strain, a workflow based on previously established protocols (Suchland et al., 2009; Marti et al., 2017) was developed, as illustrated in Figure 1, briefly outlined in this section and described in detail below.

In summary, TetR/RifR donor and TetS/RifR recipient strains were prepared first by re-analyzing the Tet-island of the donor strains followed by generation of RifR recipient strains. In order to identify mutations responsible for resistance to rifampicin/rifamycin, single-nucleotide polymorphism (SNP) analysis as well as 3D protein modeling of the *rpoB* gene comparing the recipient with its original strain were performed.

Next, recombinants were generated by co-culturing two parental strains, a donor and a recipient (mating pair), for multiple passages, both in the presence and absence of subinhibitory concentrations of tetracycline. Following simultaneous inoculation of both strains, recombinants were selected, using both tetracycline and rifamycin or rifampicin. The mating pairs used in this study are listed in Table 2.

**Table 2:** TetR/TetS mating pairs used in this study.

**Mating pair**	**Donor strain**	**Recipient strain**	**Whole-genome analysis**
1: SWA-141/S45 RIF	SWA-141	S45 RIF	Yes, *n* = 5, Conditions^*^ 2, 4
2: SWA-141/94 Ry	SWA-141	94 Ry	Yes, *n* = 9, Conditions 1, 2, 4
3: SWA-141/111 Ry	SWA-141	111 Ry	No
4: SWA-107/94 Ry	SWA-107	94 Ry	Yes, *n* = 3, Condition 2
5: SWA-107/111 Ry	SWA-107	111 Ry	No
6: SWA-110/94 Ry	SWA-110	94 Ry	No
7: SWA-110/111 Ry	SWA-110	111 Ry	Yes, *n* = 2, Condition 4

* *Conditions are described in detail in the subsection “co-culture experiments”*.

After two passages in selective antibiotics, a plaque assay was performed for each mating pair to obtain single clones of putative recombinants as described (Marti et al., 2017), which were identified using standard polymerase chain reaction (PCR) assays that target the *tet*A(C) gene as well as strain-specific regions. Suspected recombinants were confirmed with a stability assay based on a previously established protocol (Marti et al., 2017), and the above-mentioned PCR methods.

Finally, a limited number of recombinants were prepared for whole-genome sequencing using the Illumina MiSeq platform (Table 2). Reads were *de novo* assembled for all recombinants, annotated and mapped against their parental strains (Table 1). Subsequently, donor-derived sequences (recombinant regions) were identified in the recombinant strains using the Harvest software tool Gingr (Treangen et al., 2014). Next, recombinant regions were compared against each other regarding their size and location. Detailed protocols are listed below or can be found in the Supplementary Data section.

### Preparation of Donor Strains

Donor strains were chosen based on their Tet-island structure as determined in a previous study (Seth-Smith et al., 2017) and are listed in Table 1. The Tet-islands of the donor strains SWA-107, SWA-110, and SWA-141 were annotated based on published data (Seth-Smith et al., 2017), the Tet-island sequence of *C. suis* R19 (Accession No. AY428550.1) as well as by comparing the open reading frames (ORFs) suggested by the “Find ORFs” function of the Geneious Prime software (version 2019.2.3, Biomatters, Ltd., Auckland, New Zealand) against the NCBI database using BLASTn and further analyzing the translated sequences with the protein-protein BLAST function (plastp), using default settings (https://blast.ncbi.nlm.nih.gov/Blast.cgi; Figure 1, Step 1). Donor-specific Tet-island annotations were then transferred to their corresponding recombinants following MAFFT Alignment. Stocks were prepared as described above (“*Chlamydia* strains”).

### Generation of Rifampicin and Rifamycin Resistant *C. suis* Strains

In order to generate rifampicin and rifamycin resistant recipient strains, the strains S45, SWA-94 and SWA-111 (Table 1) were grown in increasing concentrations of rifampicin (S45) or rifamycin (SWA-94, SWA-111) for multiple passages until resistance to these antibiotics could be detected as previously described for other chlamydiae (Kutlin et al., 2005; Suchland et al., 2009), starting with a multiplicity of infection (MOI) of 2 and a subinhibitory concentration of 0.0001-0.001 μg/ml and 0.03-0.125 μg/ml for rifampicin and rifamycin, respectively (Figure 1, Step 2). Once cultures grew at previously inhibitory concentrations, resistance was suspected and stocks were prepared as described above and quantified using titration by sub-passage (Supplementary Data 1). The minimal inhibitory concentration (MIC) of the recipient strains was determined and compared to the original (S45, SWA-94, SWA-111) as well as the donor TetR strains (SWA-107, SWA-110, SWA-141) by infecting confluent cell monolayers with an MOI of 0.5, centrifugation (1,000 g, 25°C, 1 h) and replacement of the inocula with serial 2-fold dilutions of the antibiotic and comparing it to the control, as described (Wanninger et al., 2016).

In parallel, the same assay was used to determine a threshold concentration, which was defined as the concentration where the majority of the donor strain inclusions were reduced without hampering the inclusion size and morphology of the recipient strains. Finally, the stability of the resistance was tested by propagation of the strains in the absence of rifampicin (S45 RIF) or rifamycin (94 Ry, 111 Ry) for up to five passages prior to challenge with the threshold concentration to ensure that the resistance is stable and that the threshold concentration can be used for selection.

### *rpoB* Sequence Analysis and 3D Protein Modeling

Next, in order to identify mutations that confer rifampicin/rifamycin resistance, whole-genome sequencing (WGS) of the recipient strains was performed followed by SNP analysis and 3D protein modeling of the *rpoB* gene comparing the recipient with their corresponding original strains as listed in Table 1 and Figure 1, Step 3. Detailed protocols for WGS are listed below in the subsection “Propagation of recombinants, tetracycline resistance screen and whole-genome analysis.” SNPs were calculated using the Geneious variant finder by comparing the original strains to the rifampicin/rifamycin-resistant recipient strains, which then generated an Excel file listing all detected SNPs.

For *rpoB* gene analysis, nucleotide sequences were extracted from the whole genomes before they were translated into amino acid sequences, and Clustal Omega Alignment was performed. Sequence analyses were performed with the Geneious Prime software and were further used for 3D protein modeling, which was performed as published previously (Somboonna et al., 2019), with modifications. Briefly, amino acid sequences for each strain were submitted to the UCSF online platform MODBASE with the settings “best scoring model,” “longest well-scoring model,” and the “Slow (Seq-Prf, PSI-BLAST)” Fold assignment method (https://modbase.compbio.ucsf.edu/modweb/; Eswar, 2003; Pieper et al., 2014). The PyMOL software (The PyMol Molecular Graphics System, Version 1.2r3pre, Schrödinger, LLC., New York, NY, USA) was then used to visualize, interpret and curate the models comparing the original strain with the RifR recipient strain.

### Co-culture Experiments

First, SPG stocks of the TetR donor and the TetS recipient strains were added together (co-culture) or separately (parental strain controls) to cycloheximide-free infection medium at a MOI of 0.1 and 0.5 per ml medium, respectively. The three suspensions (TetR-only, TetS-only, Mix) were used to inoculate confluent monolayers (200,000 cells) with 1 ml per well. In total, each suspension infected four wells as shown in Figure 1 (Step 4).

Plates were then centrifuged for 1 h at 1,000 g and 25°C. Following centrifugation, inocula were replaced with either infection medium (Condition 1; C1; no Tet) or infection medium containing subinhibitory concentrations of tetracycline (1/2 MIC of the TetS strain; Condition 2). After 48 h of incubation (37°C, 5%), cultures were scraped and used to infect fresh monolayers. Following centrifugation (1 h, 1,000 g, 5°C), inocula were replaced with (a) selective antibiotics (Selection 1; S1) or (b) infection medium with/without subinhibitory concentrations of tetracycline (Selection 2; S2) (Figure 1, Step 4). The four conditions for each co-culture are listed in Table 3.

**Table 3:** List of conditions used per co-culture experiment.

**No**.	Name^*^	**Tetracycline culture (Condition C)**	**Tetracycline passage (Selection S)**
1	C1S1	No tetracycline in culture medium	Tetracycline selection added at Passage 1
2	C1S2	No tetracycline	Selection at Passage 2
3	C2S1	Subinhibitory tetracycline	Selection at Passage 1
4	C2S2	Subinhibitory tetracycline	Selection at Passage 2

* *C, condition; S, selection*.

### Plaque Assay

After another incubation period of 48 h, cultures were passaged and replaced with selective antibiotics (all conditions). Three days after this final passage (72 h), single-infected conditions were fixed in chilled methanol for immunofluorescence assay (IFA, Supplementary Data 1) and co-cultures were inoculated onto 6-well plates to perform a plaque assay according to a previously established protocol (Marti et al., 2017) with minor modifications. In brief, 6-well plates were seeded, incubated until the monolayers were confluent and inoculated with cultures from one of the four above-mentioned conditions followed by a 10-fold dilution series (Figure 1, Step 5). After centrifugation as above and an incubation period of 24 h, infected wells were overlaid with 11% Seakem ME agarose (Lonza, Basel, Switzerland) and mixed 1:1 with cycloheximide-free infection medium. After 12–24 h of infection (hpi), 12 visible inclusions were picked and transferred to a 24-well plate where picks were cultured up to six times in confluent monolayers of a 24-well plate. The detailed protocol is listed in Supplementary Data 2. Successfully picked and grown cultures (picks) were aliquoted and frozen in SPG at −80°C until further use.

### Identification of Recombinants

Identification of recombinants was divided into two steps: (a) PCR detection and (b) final confirmation by repeated selection and stability assay (Figure 1, Step 6). For PCR detection, 100 μl of *Chlamydia*/cell suspension was extracted using the DNeasy Blood & Tissue Kit (Qiagen, Hilden, Germany) and then subjected to three conventional PCRs per successful pick: (a) the *tetA*(C) PCR to detect the presence of the Tet-island, and strain-specific PCRs for both the (b) donor and the (c) recipient strain used in the co-culture experiment.

The *tetA*(C)-specific PCR was designed previously (Dugan et al., 2004) and used according to established protocols (Wanninger et al., 2016) with modifications. Strain-specific PCRs were designed and established in-house. For the design, regions were selected that were both genetically variable and located at least 300 kbp up- or downstream of the Tet-island to reduce the risk of missing putative recombinants. The gene encoding for the polymorphic membrane protein B (*pmpB*), located ~700 kbp downstream of the Tet-island, was the most commonly used target for all donor and recipient strains except for strains S45 (intergenic region between *pmpB* and *pmpC*) and SWA-107 (plasticity zone gene *pld 6*, ~380 kbp downstream of the Tet-island). A recipient strain-specific PCR with the primer pair SWA-TS_1F/SWA-TS_1R was also created. Detailed PCR protocols and primer sequences are listed in the Supplementary Table 1A. PCR results allowed the categorization of successful picks into four categories: putative recombinant, mixed infection, Tet-island negative culture and other cultures (Supplementary Table 1B).

Putative recombinants and mixed infections were further processed with two additional passages in selective antibiotics. Negative cultures were discarded, while successfully grown cultures were collected for further testing. Again, DNA from additionally selected cultures was tested with the same conventional PCRs as above. Only putative recombinants were then further analyzed by stability assay where they were passaged in the absence of selective antibiotics for five to ten passages, (a) analyzed by PCR after Passage 0, 5, and 10 and (b) subjected to an antibiotic susceptibility assay *in vitro* following Passages 5 and 10 (Supplementary Figure 1). Cultures that were *tetA*(C)-positive, donor-negative, and recipient-positive after every tested passage as well as resistant to both tetracycline and rifamycin or rifampicin were defined as confirmed recombinants, while all other cultures were discarded.

### Propagation of Recombinants, Tetracycline Resistance Screen, and Whole-Genome Analysis

A limited number of confirmed recombinants (*n* = 19) comprising four separate experiments, four mating pairs and three conditions were further processed for WGS (Table 2 and Figure 1, Step 7). Confirmed recombinants were propagated in cell culture in cycloheximide-free infection medium containing selective antibiotics to produce SPG stocks using the same methods as described above. As a final confirmation to ensure that the Tet-island was not lost during stock preparation, a tetracycline resistance screen (Tet screen) was performed as described (Marti et al., 2018). In brief, each recombinant stock was used to infect two seeded ∅ 13 mm coverslips (Thermo Scientific) in a 24-well plate at a MOI of 0.5. Following centrifugation (1 h, 1,000 g, 25°C), inocula were removed and replaced with fresh cycloheximide-containing medium with (Tet) and without (control) 0.5 μg/ml tetracycline. After 48 hpi, the coverslips were fixed in methanol and IFA was performed (Supplementary Data 1). Recombinant stocks were considered resistant to tetracycline if the inclusion size and number was comparable between treated monolayers (Tet) and the untreated control. They were considered sensitive to tetracycline if the inclusion size and number on the treated monolayer was visibly reduced compared to the control. Original SWA-94 stock was used as a negative and SWA-141 as a positive assay control. Only TetR stocks were further processed for WGS. Sequencing of samples was performed at the Functional Genomics Center Zurich (FGCZ) on the Illumina MiSeq platform with 150 base pair (bp) paired-end (PE, 25M) reads output following Illumina TrueSeq Nano DNA Library preparation according to manufacturer's instructions.

Raw sequences were then trimmed, corrected and *de novo* assembled using SPAdes (Bankevich et al., 2012), which resulted in multiple contigs. Pseudo contigs were created following alignment of the shotgun contigs against the S45 genome using CONTIGuator (Galardini et al., 2011). Finally, pseudo contigs were annotated using the PROKKA software (Seemann, 2014) with S45 as the reference, resulting in annotated genome sequences of the recombinants.

**Figure 2:**
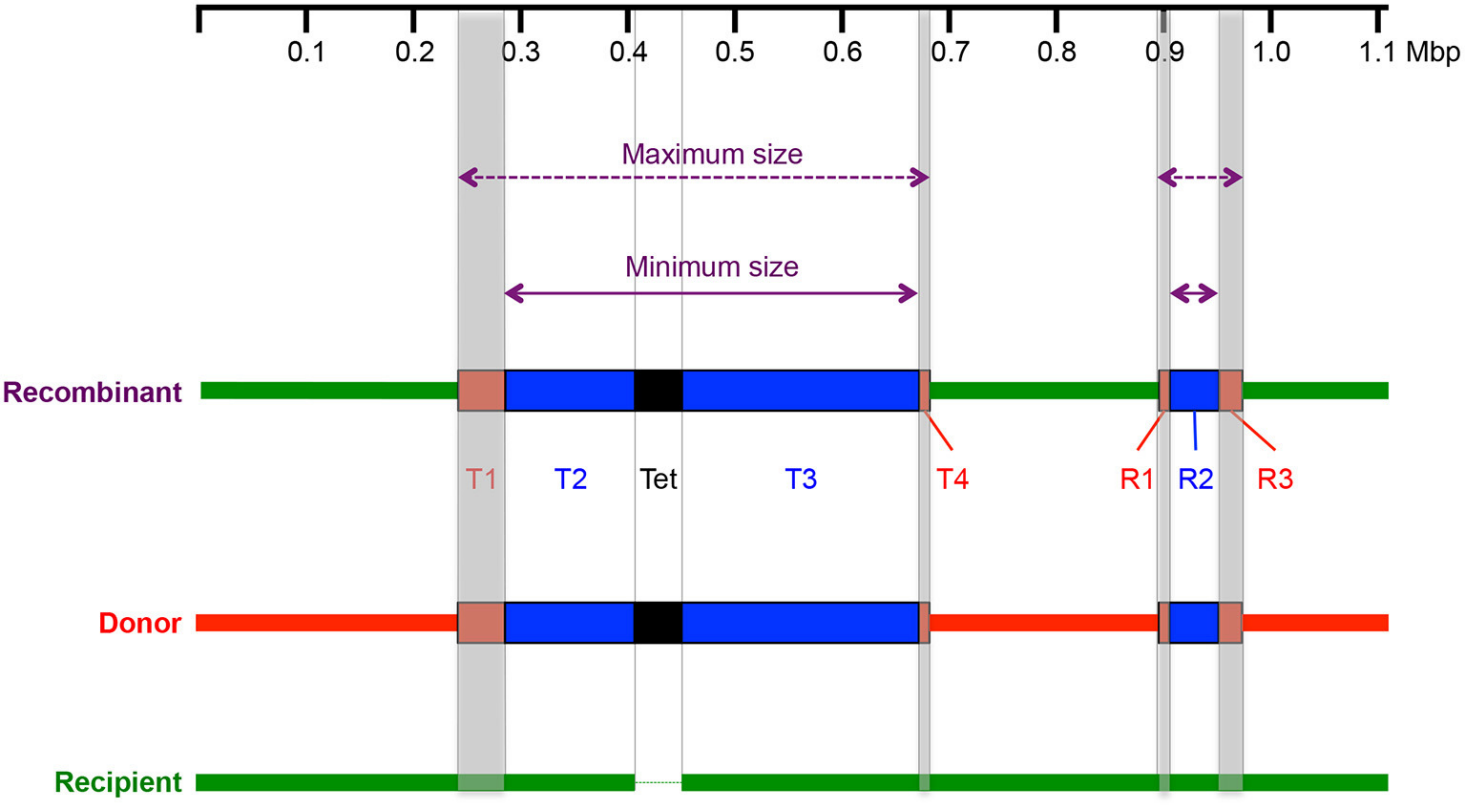
Identification of donor–derived sequences with (Tet–insert) and without (insert) the Tet–island using Gingr analysis. Shown is an artificial recombinant (purple), which consists of the recipient backbone (green) and donor-derived sequences (red and blue boxes) including the Tet-island (black, Tet). In this example, there is one Tet-insert (T1–T4) and one regular insert (R1–R3). Gray lines depict the coordinates used to identify the inserts. The coordinates of the blue blocks represent the first mismatch between the recombinant and the recipient genome upstream or the last mismatch between the recombinant and the recipient genome downstream; thus, these blocks represent the minimum size of the insert (purple arrows, solid lines). Shaded regions (T1, T4, R1, R3) could be both donor- or recipient-derived because both parental strains and the recombinant are homologous in this region. Therefore, the maximum coordinates of the red blocks were either the last mismatch between the donor and the recombinant sequence upstream or the first mismatch between donor and recombinant downstream and represent the maximum insert size (purple arrows, dashed lines). In this example, the Tet-insert is 0.38 Mbp (minimum insert) to 0.42 Mbp (maximum insert) long, while the regular insert is ~0.05–0.08 Mbp long.

Recombinant sequences were mapped against the donor and the recipient with the Harvest suite (https://harvest.readthedocs.io/en/latest/) containing Parsnp alignment and the visualization tool Gingr (Treangen et al., 2014), from where donor-derived sequences were identified with the help of mismatches between the recipient and the recombinant sequence, which were denoted as either a regular insert or a Tet-island-containing insert (Tet-insert) (Figure 2). Regular inserts were characterized by four coordinates comprising three blocks (R1-3), of which R2 represented the minimum and R1 to R3 the maximum size of the insert. Tet-inserts are characterized by six coordinates resulting in five blocks (T1, T2, Tet-island, T3, and T4). Here, T2/Tet-island/T3 represents the minimum size of the insert, while all five blocks together represent the maximum insert size. Specifically, the minimum insert size is defined as the distance between the first recombinant/recipient genome mismatch upstream and the last recombinant/recipient genome mismatch downstream. Therefore, the maximum insert size is defined as the distance between the last recombinant/donor mismatch upstream—and the first recombinant/donor mismatch downstream.

Next, annotated recombinant genomes were saved in the GenBank format (.gbk) and uploaded to the Geneious Prime software, where recombinant insert coordinates were added as a Motif using the “Add annotation” function. All sequences were then rearranged to start at Locus tag 1 of S45 and all annotations were removed except for the assembly gaps and the recently added motifs. This step was followed by MAFFT Alignment with S45 and the transfer of all coding sequence (CDS), gene, rRNA, tmRNA, and tRNA annotations from S45 to the recombinant sequence. Finally, SNP analysis was performed as described above using the Geneious variant finder by comparing donor-derived sequences to the corresponding donor strain and recipient-derived sequences to the corresponding recipient strain.

### Statistical Analysis

Co-culture experiments including recombination efficiencies were evaluated using descriptive statistics tools followed by the chi square test of the SPSS Statistics software (version 23, IBM, Armonk, NY, USA) as well as the Fisher's exact test by GraphPad QuickCalcs to compare different conditions (https://www.graphpad.com/quickcalcs/contingency1/). Single comparisons were considered significant at a *p* ≤ 0.05 (two tailed), whereas the Bonferroni correction was applied for multiple comparisons where the significant *p*-value was determined by dividing 0.05 by the number of comparisons performed (Haynes, 2013).

Graphpad Prism (v. 8, https://www.graphpad.com/scientific-software/prism/) was used for all statistics involving recombinant analysis using either the One-way ANOVA (Kruskal-Wallis test) with Dunn's multiple comparisons test for multiple means, or the Mann-Whitney test for the comparison of two means. A *p* ≤ 0.05 was considered significant unless otherwise mentioned in the text.

## Results

### SWA-141 Possesses the Complete Tet-Island, While Both SWA-107 and SWA-110 Are Truncated

Donor strains were chosen based on the structure and size of their Tet-island, which ranged between 7.3 and 12.1 kb in length: SWA-141 possesses the full-length Tet-island (12,107 bp) and is identical to that of the TetR type strain R19 (Dugan et al., 2004; Dimond and Hefty, 2020), while SWA-107 (9,699 bp) has a partial deletion in the *inv* gene and in the *mob* gene region. SWA-110 (7,330 bp) has identical deletions and further does not possess the two *IScs605* transposases, which in turn led to a fusion of the *tetR*(C) repressor with the *inv* gene, as described (Seth-Smith et al., 2017).

**Figure 3:**
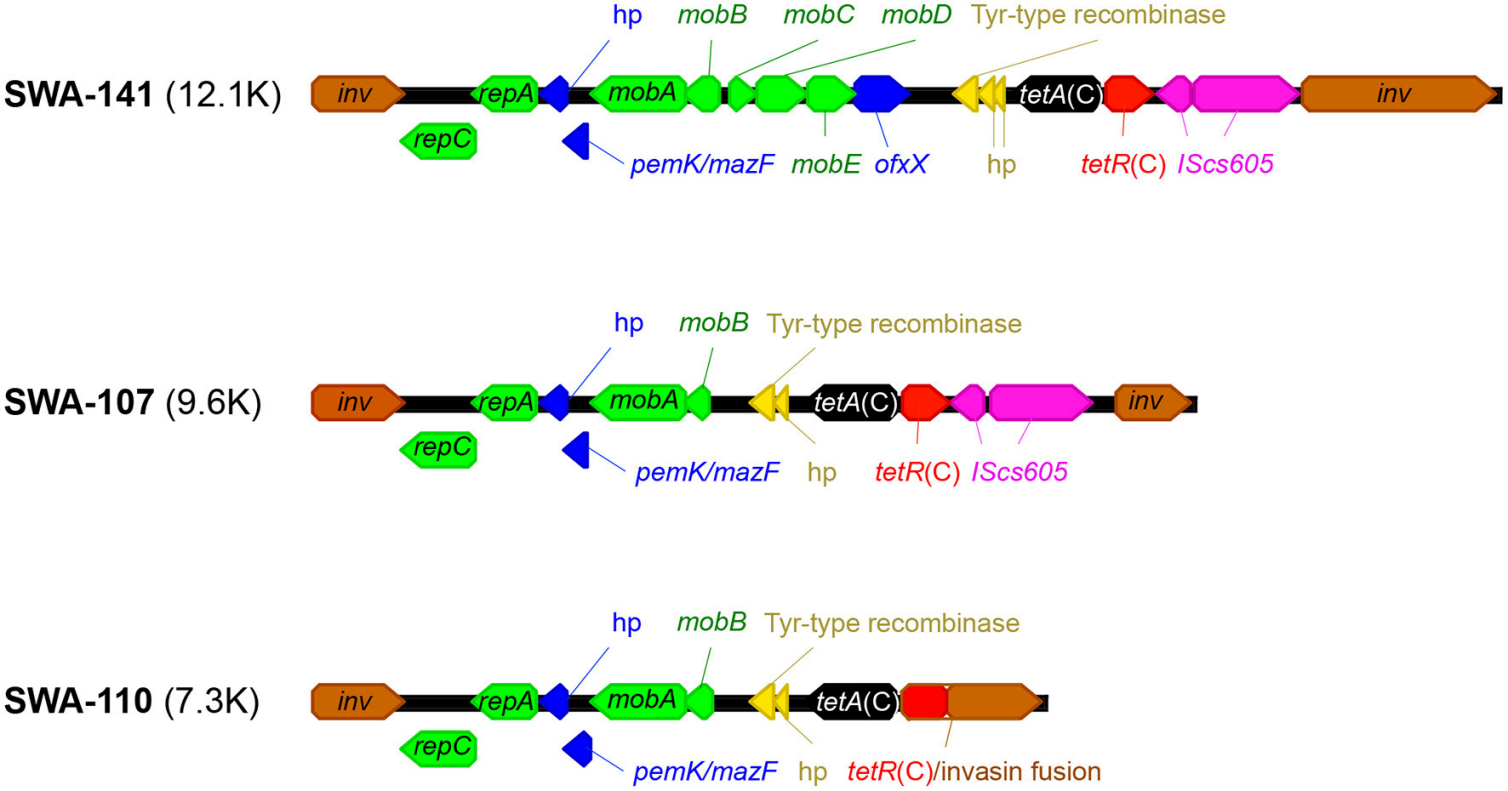
Tet-island structure of donor strains. Shown is the updated structure of the Tet-islands of the *C. suis* donor strains SWA-141, SWA-107, and SWA-110, as described previously (Seth-Smith et al., 2017). The *inv* gene is depicted in brown, the *mob* and *rep* genes in green, *pemK/mazF* and its neighboring hypothetical protein in blue. Moreover, the newly identified tyrosine-type recombinase/integrase and neighboring hypothetical proteins are shown in yellow, the *tetA*(C) in black, *tetR*(C) in red and the transposases of the insertion element IScs*605* in pink. The *tetR*(C)-*inv* fusion protein of SWA-110 is labeled “*tetR*(C)/invasin fusion” with the gene-specific coloring left intact.

Detailed analysis of the Tet-island using the “Find ORFs” tool on Geneious and subsequent BLASTp analysis revealed two to three very short putative CDSs between the *mob* genes and *tet*A(C), of which one (SWA-107, SWA-110) or two (SWA-141) were hypothetical proteins of around 60 amino acids (aa) in length, and a 106 aa long protein, which shared 100% amino acid identity (AAI) with “MULTISPECIES: tyrosine-type recombinase/integrase [Bacteria]” (query cover: 95%; Accession No. WP_009873361.1, Figure 3: indicated in yellow), a lambda (λ) integrase (Guo et al., 1997; Kwon et al., 1997). Moreover, a hypothetical protein with 119 amino acids was identified in all three strains between *repA* and *mobA*. The updated structure of the Tet-island for all three strains is shown in Figure 3 and the Tet-island sequences with annotations are listed in Supplementary Table 2.

Finally, the minimal inhibitory concentration (MIC) of all three strains was determined in order to establish a selection concentration for tetracycline. All three strains have a minimal inhibitory concentration (MIC) of 4 μg/ml to tetracycline as determined by antibiotic susceptibility assay according to previously established methods (Wanninger et al., 2016; Marti et al., 2018). For selection in co-culture experiments, 1 μg/ml of tetracycline was used as all TetS strains used in this study have MICs to tetracycline ranging between 0.03 and 0.0125 μg/ml (Wanninger et al., 2016), and the TetR strains are largely unaffected at concentrations up to 1 μg/ml (Marti et al., 2018).

### Resistance to Rifampicin/Rifamycin Was Induced After ~10–30 Passages in Subinhibitory Concentration of the Drug

Strains S45, SWA-94, and SWA-111 were cultured in subinhibitory concentrations of either rifampicin or rifamycin until they developed resistance against these antibiotics as previously described for *C. trachomatis* and *C. pneumoniae* (Kutlin et al., 2005). After 10–11 passages, S45 developed low-level resistance to rifampicin with a new MIC of 0.03 μg/ml corresponding to an 8-fold resistance increase (Table 1). For SWA-94 and SWA-111, rifamycin instead of rifampicin was used because SWA-94 was highly sensitive to rifampicin (MIC: 0.00024 μg/ml) and the resulting 8-fold resistance increase (0.002 μg/ml) did not allow distinctive selection from the TetR donor strain SWA-141 (0.001 μg/ml). Rifamycin resistance emerged after 18 and 29 passages for SWA-94 and SWA-111, respectively, which then led to an 8- to 32-fold resistance increase (Table 1).

After SPG stocks were made for future co-culture experiments, the stability of the resistance was confirmed and the appropriate selection concentration determined. For the former, the RifR strains, now termed S45 RIF, 94 Ry, and 111 Ry, were passaged five times in the absence of antibiotics before the MIC was determined (data not shown). Next, the MICs of the RifR strains to rifampicin/rifamycin were compared to that of the TetR strains in order to determine an appropriate concentration for selection. The MIC of the donor strains ranged between 0.0005–0.001 μg/ml for rifampicin and 0.5–1 μg/ml for rifamycin resulting in a selection concentration of 0.015 μg/ml for rifampicin (experiments with S45 RIF) and 1 μg/ml for rifamycin (94 Ry, 111 Ry).

### Rifamycin Resistance in Recipient Strains Was Likely Caused by Mutations in the *rpoB* Gene

In order to identify changes linked to RifR resistance, the genomes of the recipient strains were sequenced and compared against the original strain (accession numbers in Table 1). In total, 74, 13, and 19 SNPs were detected in S45 RIF, 94 Ry, and 111 Ry, respectively, of which 35, 11, and 14 SNPs had an effect on a protein (Supplementary Table 3). Regarding resistance to rifamycin or rifampicin, the most notable changes in all three strains were non-synonymous mutations in the *rpoB* gene encoding for the β-subunit of the bacterial RNA polymerase (RNAP), which in turn is the target structure of the rifamycin group. These mutations are known to confer antibiotic resistance in various bacteria (Rabussay and Zillig, 1969; Jin and Gross, 1988; Campbell et al., 2001) including *Chlamydia* (Suchland et al., 2005). There were seven clade-specific, non-synonymous mutations separating the recipient from the donor strains (Recipient/Donor mutation: V157M, V242T, V275I, I668V, V910L, E974D, V1249L, Supplementary Table 4). Moreover, each of the recipient strains had one amino acid change that did not correspond to clade-specific changes.

**Figure 4:**
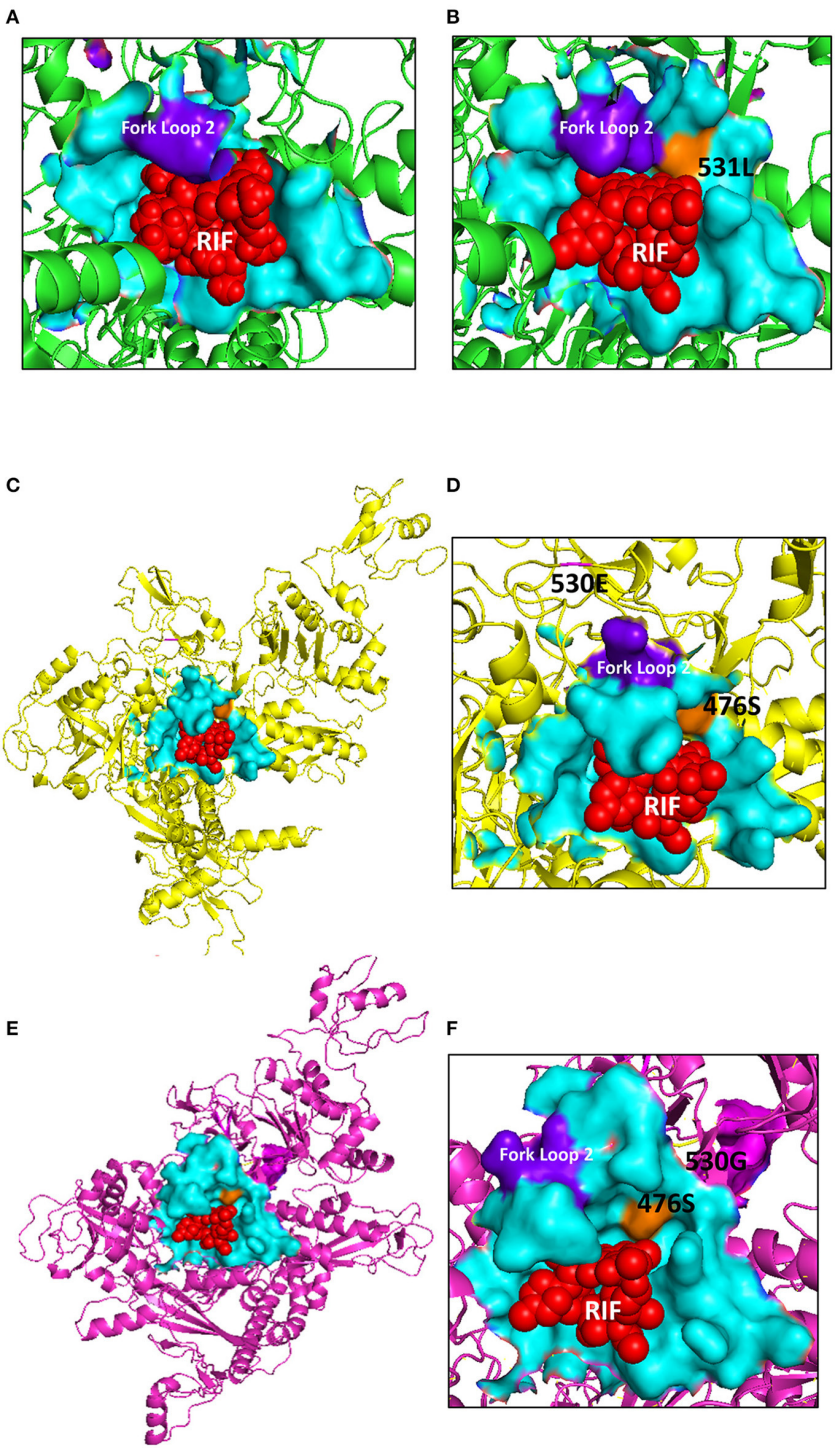
3D modeling of the RpoB protein of *C. suis* S45 and rifampicin (RIF) resistant S45 compared to *E. coli* RNA polymerase (RNAP) with RIF complex (RpoB S531L mutant 5UAL). **(A)** The RIF binding sites of *E. coli* 4KMY wildtype RNAP.RFP complex; **(B)** the *E. coli* S531L RNAP. RFP complex; **(C)** predicted 3D structure of *C. suis* S45 G530E RIF resistant RpoB protein; **(D)** RIF binding sites of *C. suis* S45 RIF resistant G530E RNAP. RFP complex; **(E)** predicted 3D structure of *C. suis* S45 wildtype RpoB protein; and **(F)** RIF binding sites of *C. suis* S45 wildtype RNAP. RFP complex. The RIF binding pocket is shown in cyan (surface models), the fork loop 2 in purple (surface models), the S531L mutation is shown in orange (surface models), the 530E and 530G residues in *C. suis* S45 RIF and S45 are shown in magenta and yellow (cartoon models) and RIF in red (sphere models).

Specifically, S45 RIF has glutamic acid (E) in codon 530 as opposed to the glycine (G) present in its parent and the other recipient/donor strains (G530E mutation; Table 1 and Supplementary Tables 3-1, 4-1). The sequences of the *rpoB* gene of both S45 and S45 RIF (1252 amino acids) were submitted to the ModWeb modeling pipeline resulting in a Model score of 1 (E-value: 0) with 1245 amino acids (position 3–1247) sharing 47% sequence identity (8–1339) with the *Escherichia* (*E*.) *coli* RNAP and rifampin complex, *rpoB* S531L mutant (PDB: 5UAL). This mutant possesses a disoriented fork loop 2, which is crucial for interaction between the RNAP of *E. coli* and rifampin, as well as an S541L mutation in the binding pocket of 5UAL that is known to cause resistance to rifampin (Molodtsov et al., 2017; Figure 4A). 3D structures were then visualized using the PyMOL software revealing that both the original S45 and the recipient strain S45 RIF have a disoriented fork loop 2. Interestingly, the individual structures of the fork loop 2 are not identical for S45 and S45 RIF. Moreover, while the G530E mutation, which corresponds to the codon 585 of 5UAL (Supplementary Tables 4-1), is located close to the rifampin binding pocket, it is not located within the pocket (Figure 4). Moreover, as listed in Supplementary Table 2, there was an insertion (tandem repeat, *n* = 5 instead of *n* = 4 adenosines, S45 position: 950987-8) leading to a frame shift mutation in *rpoC1* and *rpoC2*. However, Sanger sequencing of the region did not find any difference between the original S45 and S45 RIF. Furthermore, comparison of all the recipients and their original as well as the donor strains yielded five adenosines in this region resulting in an intact *rpoC* gene instead of the two truncated *rpoC1* and *rpoC2* as annotated in S45, indicating that there was a sequencing error for S45 in this region rather than a true insertion (data not shown).

In contrast, both 94 Ry and 111 Ry had an identical non-synonymous mutation in codon 461 changing the aspartic acid to an alanine (D461A; Table 1 and Supplementary Tables 3, 4), a binding site for rifampicin/rifamycin (Suchland et al., 2005) and known to confer resistance (Goldstein, 2014).

### The Overall Recombination Efficiency of *C. suis* Co-culture Experiments Was 28.0%

Following co-culture and selection, plaque assays were performed as described in the Material and Methods section. As observed in previous studies (Marti et al., 2017), *C. suis* does not form visible plaques. Inclusions had to be picked blindly following a brief check by light microscopy to confirm the presence of chlamydial inclusions (Supplementary Data 2). Overall, 12 plaque assay picks were obtained per condition (*n* = 48) from each of the 21 co-culture experiments resulting in a total of 1,008 picks, of which 812 grew successfully, corresponding to a plaque assay-related growth success rate of 80.6%.

Initial identification of recombinants by PCR of the 812 remaining cultures resulted in 311 putative recombinants (311/1,008, 30.9%), 175 mixed infected cultures (175/1,008, 17.4%), 293 Tet-island negative cultures (293/1,008, 29.1%), and 33 cultures with ambiguous PCR results (“Other culture,” 33/1,008, 3.3%). All Tet-island positive cultures (*n* = 519) were then subjected to another round of antibiotic selection using tetracycline and rifampicin or rifamycin for two passages prior to repeated PCR, of which 378 cultures survived repeated selection (378/1,008, 37.5%).

Finally, following identification of recombinants by additional selection, PCR and plaque assay, 282 of all picks were confirmed as recombinants resulting in an overall recombination efficiency of 28.0%.

### Subinhibitory Concentrations of Tetracycline Inhibited the Recombination Efficiency *in vitro*

One primary aim of this study was to optimize co-culture protocols and to determine factors that may increase recombination efficiency. These factors included: (a) the growth protocol itself where antibiotic selection was either applied sequentially or simultaneously; (b) subinhibitory concentrations of tetracycline during co-culture (Condition C); and (c) an additional passage after co-culture prior to selection with tetracycline (Selection S). A flow diagram illustrating these growth protocols and factors is shown in Supplementary Figure 2.

To compare growth protocols, the first four co-culture experiments (*n* = 192 picks) were performed with sequential selection. Specifically, cultures were treated with either rifampicin/rifamycin or tetracycline in the first passage followed by exchanging the antibiotics in the second passage prior to plaque assay. In six subsequent co-culture experiments using the same mating pairs, selective antibiotics were applied simultaneously (*n* = 288). The recombination efficiency was significantly higher (*p* < 0.0001) in the co-culture experiments under simultaneous selective pressure (134/288, 46.5%) compared to the sequential selection protocol (30/192, 15.6%).

Additionally, all co-cultures were performed without (C1) and with (C2) the presence of subinhibitory concentrations of tetracycline and we compared the overall recombination efficiency between these two conditions. Specifically, the concentrations used for this condition was a quarter of the tetracycline MIC (MIC_1/4_), namely 0.015 μg/ml for S45 RIF and 94 Ry, and 0.0039 μg/ml for 111 Ry. The recombination efficiency of the control condition was significantly higher (166/504, 32.9%) compared to the tetracycline condition (116/504, 23.0%) with a *p*-value of 0.0086.

For the third factor of interest, tetracycline selection was performed immediately at the first passage following co-culture (S1) or after a selection-free passage (S2). In detail, the first passage at 48 h post co-culture was either performed in medium with tetracycline as the selective antibiotic (no-passage condition, S1) or in medium (passage condition, S2) with (sequential protocol) or without rifamycin (simultaneous protocol) before they were processed in parallel. Here, the passage condition yielded significantly more recombinants (177/504, 35.1%) compared to the no-passage condition (105/504, 20.8%) with a *p*-value of 0.0002 over all co-culture experiments, indicating that tetracycline inhibited the recombination efficiency.

### Co-cultures With SWA-110 Have a Significantly Reduced Recombination Efficiency

**Figure 5:**
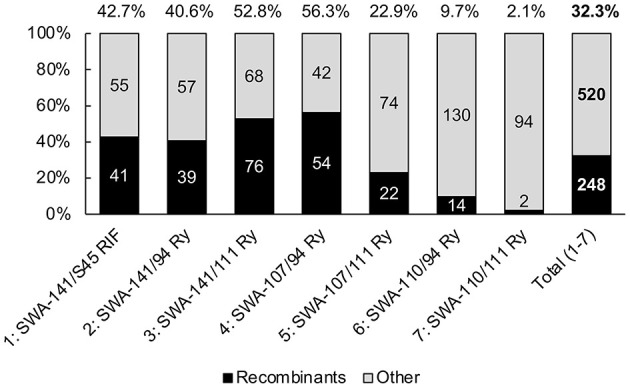
Recombination efficiencies following simultaneous selection. Shown are the recombination efficiencies for each mating pair (1–7) in a compound bar chart. The bars represent 100% of picks per mating pair, of which the black section represents the absolute number of confirmed recombinants and the gray section represents all other picks (mixed culture, parent-only culture). The recombination efficiency per mating pair (in %) is shown on top of each bar.

As a next step, the influence of the seven mating pairs on the recombination efficiency was evaluated. To reduce confounding influences from the growth protocols used, only 16 co-culture experiments with a total of 768 picks resulting from simultaneous selection were used for analysis. The average recombination efficiency across all mating pairs using the growth protocol with simultaneous selection was 32.3% (Figure 5), but there were significant differences between mating pairs as determined by the chi-square test. Subsequently, the recombination efficiencies of the different mating pairs were compared using the Fisher's exact test (Supplementary Table 5).

While there was no difference between co-culture experiments involving SWA-141 and SWA-107, experiments with SWA-110 yielded significantly fewer recombinants compared to all other co-culture experiments. Moreover, SWA-107/111 Ry yielded fewer recombinants than SWA-107/94 and SWA-141/111 Ry.

In summary, while all other donor and recipient strains used in this study yielded overall comparable recombination efficiencies, matings with SWA-110 resulted in significantly fewer recombinants. For SWA-110/111 Ry, two co-culture experiments (no. 12, 16) were performed yielding only two recombinants for exp. 12 and none for exp. 16 (2/96, 2.1%; Figure 5). An additional co-culture experiment was then performed using five times higher chlamydial concentrations with MOIs of 0.5 and 2.5 for SWA-110 and 111 Ry, respectively, in order to produce additional recombinants for whole-genome analysis. The resulting recombination efficiency was 8.3% (4/48), which was not significantly higher than the original protocol (*p* = 0.1829).

### Recombinants Obtained From the Same Plaque Assay May Be Sibling Clones

**Figure 6:**
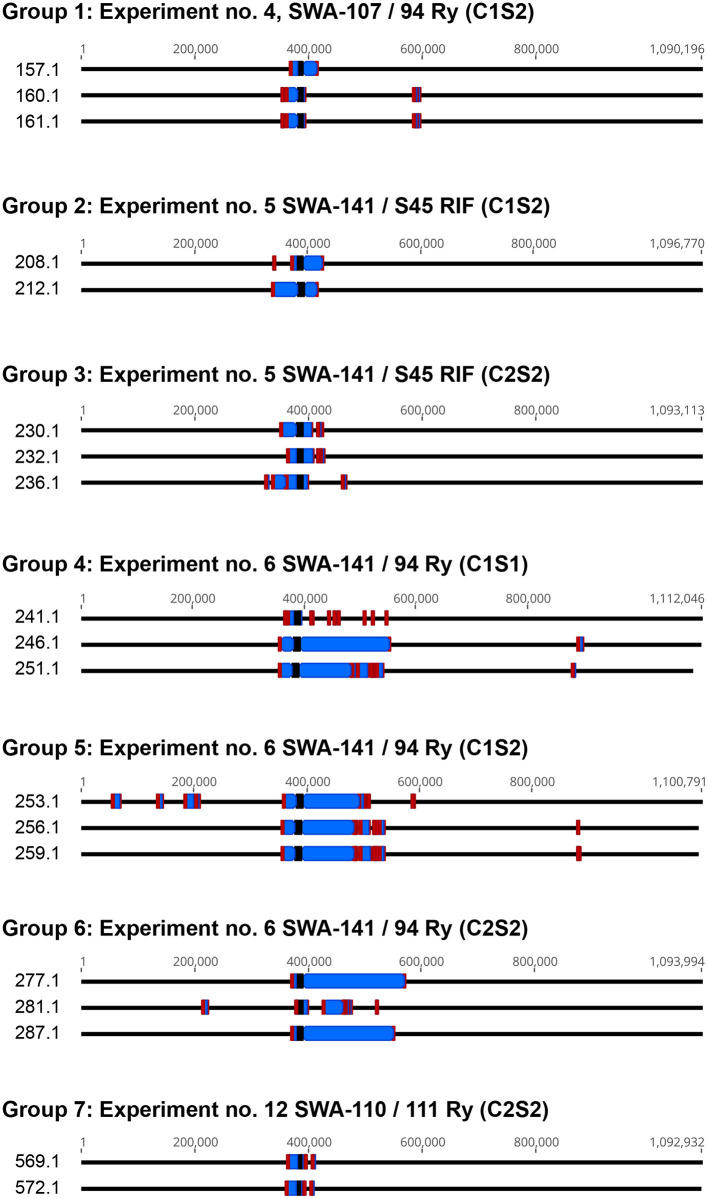
Overview of recombinants obtained from the same experiment and co-culture condition. Shown are sequenced genomes of the nineteen recombinants that were sequenced in parallel of one or two cultures obtained from the same plaque assay. Chromosomes were linearized and donor-derived sequences are labeled in red (homologous region spanning the maximum and minimum mismatch between recombinant and the recipient strain), blue (donor-derived sequences) and black (Tet-island). Images were created with Geneious (v. 2019.2.3).

Following confirmation of 282 recombinants obtained from the sequential and the simultaneous co-culture protocol, four different culture conditions (C1S1, C1S2, C2S1, C2S2) and seven mating pairs, we aimed to evaluate whether cultures that were grown from the same plaque assay and therefore the same co-culture experiment and condition resulted in an increased number of sibling clones as suggested in our preliminary study (Marti et al., 2017). In order to evaluate this, nineteen recombinants from four co-culture experiments and seven separate plaque assays (groups) were first screened to ensure tetracycline resistance (Supplementary Figure 3) followed by whole-genome sequencing (Figure 6). Specifically, experiments no. 4, 5, 6, and 12 were chosen for this analysis resulting in groups 1, 2/3, 4–6, and 7, respectively (Table 4 and Supplementary Table 6).

**Table 4:** List of groups where more than one recombinant from the same plaque assay was sequenced.

**Plaque assay group**	**Recombinant**	**Accession no**.	**Combination**	**Condition**	**Recombinant**	**Unique**
1	157.1	CP063188	SWA-107/94 Ry	C1S2	Yes	Yes
	160.1	CP063187			Yes	No
	161.1	CP063186			Yes	No
2	208.1	CP063185	SWA-141/S45 RIF	C1S2	Yes	Yes
	212.1	CP063184			Yes	Yes
3	230.1	CP063183	SWA-141/S45 RIF	C2S2	Yes	No
	232.1	CP063182			Yes	No
	236.1	CP063181			Yes	Yes
4	241.1	CP063180	SWA-141/94 Ry	C1S1	Yes	Yes
	246.1	CP063179			Yes	No
	251.1	CP063178			Yes	No
5	253.1	CP063177	SWA-141/94 Ry	C1S2	Yes	Yes
	256.1	CP063176			Yes	No
	259.1	CP063175			Yes	No
6	277.1	CP063174	SWA-141/94 Ry	C2S2	Yes	No
	281.1	CP063173			Yes	Yes
	287.1	CP063172			Yes	No
7	569.1	CP063171	SWA-110/111 Ry	C2S2	Yes	No
	572.1	CP063170			Yes	No

Of the 19 recombinants, seven were unique while 12 were identical either upstream and/or downstream of the Tet-insert. Identical sibling clones were found in Groups 1 (160.1/161.1), 5 (256.1/259.1), and 7 (569.1/572.1). Interestingly, recombinant 251.1 from Group 4 was identical to 256.1 and 259.1 from Group 5, while 246.1 (Group 4) shared upstream coordinates with these sequences (Figure 6).

### The Average Size of the Tet-Insert Ranged Between 19,510 and 198,402 bp

In order to confirm the recombinant regions, SNP analysis was performed comparing the recombinant insert region against the donor and background regions against the recipient strain genome sequence. Data for analysis of all 19 recombinants is provided in Supplementary Table 7.

Common SNPs were found in the gene encoding for the histone-like protein (*hctB*), the organic solvent tolerance protein OstA (*ostA*), the polymorphic membrane protein B (*pmpB*) and the RNAP-associated protein-encoding gene (*rapA)*. Specifically, the mating pairs SWA-141/S45 RIF, SWA-141/94 Ry, and SWA-110/111 Ry commonly yielded recombinants with SNPs in *hctB*, while *pmpB* SNPs were found in SWA-141/94 Ry and SWA-107/94 Ry. SNPs in *ostA* and *rapA* were often found in recombinants emerging from the mating pair SWA-141/S45 RIF (Table 5).

**Table 5:** Overview recombinant analysis based on mating pairs.

**Group**	**Recombinants**	**Combination**	**Common SNPs**	Insert range (bp)^*^	**Tet-insert range (bp)**
1	157.1, 160.1, 161.1	SWA-107/94 Ry	*pmpB*	1,689–3,703	29,486–45,089
2	208.1, 212.1	SWA-141/S45 RIF	*hctB, ostA, rapA*	504	53,855–77,978
3	230.1, 232.1, 236.1			519–23,495	36,647–53,396
4	241.1, 246.1, 251.1	SWA-141/94 Ry	*hctB, pmpB*	331–16,809	22,250–196,053
5	253.1, 256.1, 259.1			645–16,809	126,495–132,719
6	277.1, 281.1, 287.1			2,231–34,461	19,510–198,402
7	569.1, 572.1	SWA-110/111 Ry	*hctB*	407–2,395	26,129–26,797

* *Strains 157.1, 212.1, 277.1, and 287.1 only possessed the Tet-insert*.

All of the 19 recombinants possessed donor-derived sequences containing the Tet-island (Tet-insert, Supplementary Tables 8-1). We additionally identified 59 other regions with donor-derived sequences (insert) resulting in an average of 4 recombinant regions per sequence with a range of 1–9 recombination events per sequence (Supplementary Tables 8-2). The overall average size of the donor-derived sequences was ~23 kbp with significantly larger sequences for the Tet-insert compared to the non-Tet inserts (*p* < 0.0001) using the Mann-Whitney test. Specifically, the Tet-insert was in average 81'640 bp long (19,510–198,402 bp; Table 5). In contrast, the average size of non-Tet inserts was 4,592 bp (331–34,461 bp; Table 5). The Kruskal Wallis test, used to compare the average Tet-insert size between mating pairs, did not yield a significant result (*p* = 0.1146) indicating that the mating pair does not influence the size of the Tet-insert.

Finally, we closely analyzed the breakpoint regions of Tet-inserts found in the unique recombinants (*n* = 7) and compared them to the general breakpoint regions of the sibling clones (*n* = 12) to identify genes commonly involved at the breakpoint of the Tet-insert. While there were not enough independent samples for statistical analysis, we found that the genes *cadA* (encoding for the Cadmium-translocating P-type ATPase; located 20.1 kbp upstream of Tet-island) and *ftsK* (DNA translocase FtsK; 7.3 kbp) were involved in the upstream breakpoints of more than one independent sample with two events each (*cadA:* 246.1/251.1/253.1 and 256.1/259.1; *ftskA:* 241.1 and 277.1/287.1). Moreover, *nqrF* [Na(+)-translocating NADH-quinone reductase], located 1.3 kbp downstream of the Tet-island, included recombinant breakpoints in two separate experiments (160.1/161.1 and 569.1/572.1).

## Discussion

This study developed an advanced *in vitro* co-culture protocol to rapidly generate a large number of *C. suis* recombinants with the Tet-island of a donor strain while retaining the genomic background of the TetS recipient strain, based on modifications of previously established protocols (Suchland et al., 2009; Marti et al., 2017). Our goal was to identify factors influencing recombination efficiency and to describe and discuss the methodology in detail. Altogether, we used three different clinical *C. suis* TetR donor strains and co-cultured them with one reference and two clinical TetS recipient strains, all of which had been isolated and characterized in previous studies (Wanninger et al., 2016; Seth-Smith et al., 2017).

Based on previous data (Seth-Smith et al., 2017), we analyzed the Tet-island structure of recently isolated clinical *C. suis* donor strains, SWA-141, SWA-107, and SWA-110, of which the first possessed a complete Tet-island (12.1 kbp), while the latter two were truncated with 9.6 and 7.3 kbp, respectively. In addition to the already known and described structures such as *repA, repC, pemK/mazF, mobA-E, ofxX, tetA(C), tetR(C)*, and the two transposases *IScs605* (Dugan et al., 2004; Joseph et al., 2016; Seth-Smith et al., 2017), we identified two small hypothetical proteins of unknown function as well as a tyrosine-type λ recombinase/integrase (Tyr-type recombinase) located between the *mob* genes and the *tetA*(C). Tyr-type recombinase is found on multiple bacterial species and belongs to a large family of site-specific DNA recombinases originating from phages where it enables integration and excision of the genome from its host (Landy, 1989; Esposito and Scocca, 1997). While the importance of the *IScs605* transposases for the original integration of the Tet-island into *C. suis* (Dugan et al., 2007)—but not for intraspecies Tet-island transfer *via* homologous recombination (Marti et al., 2017)—has been demonstrated in previous studies, the significance of the Tyr-type recombinase for tetracycline resistance acquisition in *C. suis* remains to be elucidated.

To rapidly produce a large number of recombinants, rifamycin or rifampicin resistance had to be induced in the TetS recipient strains by passaging the strains at subinhibitory concentrations of the antibiotic as described previously (Kutlin et al., 2005; Suchland et al., 2009). While *C. trachomatis* only took up to six passages to develop high-level resistance to rifamycin (32,000-fold) and rifalazil (500-fold) in the study by Kutlin et al. (2005), we only achieved low-level resistance to rifampicin (8-fold) and rifamycin (8 to 32-fold) for *C. suis* after 18 and 29 passages, respectively. Interestingly, these results are more in accordance with that of the more distantly related chlamydial species *C. pneumoniae* (strain TW-183) (Joseph et al., 2016), which developed low-level resistance to rifamycin after 12 passages with an MIC increase from 0.008 to 0.25 μg/ml (32-fold) (Kutlin et al., 2005).

To further characterize the nature of rifamycin resistance, WGS of the recipients were compared to their original strains revealing that S45 RIF possessed more SNPs (*n* = 74) than 94 Ry (*n* = 13) and 111 Ry (*n* = 19). This discrepancy may be explained by the fact that the genome of S45 originated from a 1960s isolate that was passaged an undetermined number of times prior to sequencing (Joseph et al., 2016), while the S45 strain used to induce rifampicin resistance came from the same isolate but was passaged over the years in another laboratory (Schiller et al., 2004). The occurrence of chromosomal mutations following intense cultivation of *C. trachomatis* has been reported previously (Borges et al., 2015), which may, in part, explain the genomic SNP differences for *C. suis* S45 in the present study.

S45 RIF, 94 Ry, and 111 Ry displayed a single non-synonymous mutation in *rpoB* that encodes for the β-subunit of RNAP, the target structure of the rifamycin group (McClure and Cech, 1978). Various studies have shown that non-synonymous mutations in the rifampin binding pocket of *rpoB* are primarily responsible for conferring resistance (Jin and Gross, 1988; Aubry-Damon et al., 1998; Wichelhaus et al., 1999; Goldstein, 2014; Zaw et al., 2018). The binding pocket is located within the main channel of the RNAP β-subunit and interacts with the rifamycin group primarily through hydrophobic side chains as well as *via* hydrogen bonds interactions with the polar side chains of the drug (Campbell et al., 2001).

Protein modeling of the S45 RIF *rpoB* β-subunit revealed similarity to the *E. coli* rifampicin resistant S531L mutant (Figure 4). While G530E is not directly part of the rifamycin group binding pocket (Supplementary Data 14), the crystal structure revealed a disoriented fork loop 2 (residues 534–541) compared to the wild type, which is known to result in decreased binding activity between the pocket and the drug in the S531L mutant (Molodtsov et al., 2017). However, although the modeled S45 RIF protein was different from the original S45 strain in this region, it is not clear whether *C. suis* generally possesses a disoriented fork loop 2 compared to *E. coli*, thus the effect of this structural change is difficult to predict.

The glycine on codon 530 conserved in *E. coli* and *Mycobacterium tuberculosis* (codon 585) does not directly bind to rifamycin and is located between mutation clusters II and III, which are known to regularly confer rifampin resistance (Goldstein, 2014; Supplementary Data 14). While it is not generally known as an inducer of resistance to the rifamycin group in *Chlamydia* (Kutlin et al., 2005; Suchland et al., 2005) and other bacterial species (Jin and Gross, 1988; Aubry-Damon et al., 1998; Wichelhaus et al., 1999; Goldstein, 2014; Zaw et al., 2018), the G530E mutation in S45 RIF represents a significant change of the amino acid functional group replacing the non-polar glycine with the negatively charged glutamic acid. Therefore, G530E could represent a novel and unique mutation capable of conferring resistance to the rifamycin group in *C. suis*.

We further analyzed *rpoC* because compensatory mutations in that region have been observed to restore the fitness of rifamycin-resistant strains with *rpoB* mutations in *M. tuberculosis* (De Vos et al., 2013) and *Salmonella* (Brandis and Hughes, 2018). This could be an indication of *rpoB* mutation-induced rifampicin-resistance. However, the *rpoC* gene was identical for both S45 and S45 RIF. While it is possible that the rifampicin resistance in S45 RIF was conferred by a mutation in *rpoB*, we must also consider the possibility that unknown resistance mechanisms may be involved. For example, Kutlin et al. (2005) generated a highly rifamycin-resistant *C. trachomatis* strain without detecting any non-synonymous mutations in *rpoB*. In conclusion, while the significance of fork loop 2 in the β-subunit of the *C. suis* RNAP has yet to be elucidated, there is strong evidence to suggest that the G530E mutation is responsible for the resistance to rifampicin.

For both 94 Ry and 111 Ry, we detected a D461A mutation in *rpoB*. This change from the negatively charged aspartic acid to the non-polar alanine most likely caused a significant change in the structure of the amino acid functional group. Taking into consideration that codon 461 is a rifampin binding site (Supplementary Data 14; Suchland et al., 2005) and that the corresponding *E. coli* codon 516 belongs to cluster I and thus represents a hotspot for conferring rifamycin-resistance in both *E. coli* and *M. tuberculosis* (Goldstein, 2014), it is very likely that this mutation is responsible for the rifamycin resistance in both strains. Interestingly, the low-level resistance that the *C. pneumoniae* strain TW-183 developed in the study by Kutlin et al. (2005) was also conferred by a non-synonymous mutation in codon 461, although the resulting amino acid was the also negatively glutamic acid (D461E) thus the effect of this mutation remains unclear.

We determined the recombination efficiency of all mating pairs to be 28.0% in average. In contrast to extracellular bacteria, *Chlamydia* cannot be plated onto selective and non-selective agar plates with comparative enumeration of colonies. Because of this limitation, recombination efficiencies of *Chlamydia* co-culture experiments are often not reported at all (Suchland et al., 2009; Jeffrey et al., 2013) or only briefly mentioned (Suchland et al., 2019) with few exceptions (DeMars et al., 2007; DeMars and Weinfurter, 2008). In these latter studies, the authors co-cultured ofloxacin-resistant with lincomycin-, rifampin-, or trimethoprim-resistant *C. trachomatis* strains and then immediately performed comparative titration in both control and selective media after 48 hpi with subsequent determination of inclusion forming units (IFU) for the whole culture and the recombinants, respectively. Recombination efficiency/frequency was then expressed as the recombinant IFU divided by the whole-culture IFU. This likely represents the most accurate method to determine the recombination efficiency of *Chlamydia*, but has the distinct disadvantage that apparent recombination frequencies are very low (10^−4^ to 10^−3^). For the current study, we passaged cultures twice in selective antibiotics in order to enrich recombinants before a plaque assay was performed. Therefore, the recombination efficiencies reported in this study are probably the result of a considerable overestimation.

We investigated the effect of two different factors in co-culture protocols: The use of subinhibitory concentrations of tetracycline; and the effect of immediately selecting cultures with tetracycline compared to first passaging the cultures prior to tetracycline selection. We found that the simultaneous selection protocol was superior to a sequential selection protocol, which is not surprising given how well *Chlamydia* can recover from single-doses of various antimicrobial agents even at high doses (Marti et al., 2018), possibly as a result of heterotypic resistance (Suchland et al., 2003). What came as a surprise was that subinhibitory concentrations of tetracycline did not promote Tet-island transfer. Clinical studies have shown that pigs treated with tetracycline either as a prophylactic (Wanninger et al., 2016) or therapeutic (Borel et al., 2012) measure yielded a higher rate of tetracycline-resistant cultures. Moreover, in our previous work, recombinants were only obtained from co-cultures grown at subinhibitory concentrations of tetracycline (Marti et al., 2017). A possible explanation for these findings could be that bacterial growth is already reduced before the MIC is reached. Specifically, one possible way to define the MIC in *Chlamydia* is two times the concentration where over 90% of the inclusions are altered compared to the control (Suchland et al., 2003; Marti et al., 2018). Therefore, it is possible that growth of the recipient strains was inhibited prior to infection leading to a significantly lower rate of recombinants. Furthermore, although recombination promotes the development of antibiotic resistance (Perron et al., 2012), the use of antibiotics does not necessarily have a positive impact on the recombination rate of *Chlamydia* as was shown in this study. Furthermore, we found that passaging cultures one time in the absence of selective tetracycline concentrations actually increased the recombination efficiency, which appears to directly contradict the idea of selective pressure as a crucial driver of resistance. However, the most likely explanation for this observation is that recombinants are as fit as their parental strains, increase in number across the first passage and are more abundant once selective pressure is applied.

Out of nine possible mating pairs using three donor (SWA-141, SWA-107, SWA-110) and three recipient strains (S45 RIF, 94 Ry, 111 Ry), seven were applied in this study. S45 RIF derives from the laboratory strain S45, which was previously used to produce a double-resistant (ofloxacin, rifampin) *C. trachomatis* L_2_ strain recombinant (Suchland et al., 2009) and TetR resistant recombinants with an S45 genomic background (Marti et al., 2017). Our focus was on producing recombinants using the recently isolated *C. suis* recipient clinical strains 94 Ry and 111 Ry (Wanninger et al., 2016). Therefore, S45 RIF was only co-cultured with SWA-141, a strain with a complete Tet-island, as a control reaction.

We found that any combination with SWA-110 yielded significantly fewer recombinants compared to the rest. SWA-110 has a truncated Tet-island including a distinct absence of the two *IScs605* transposases suggesting that these transposases may be important not only for the initial integration (Dugan et al., 2007) but also for recombination. However, our previous study showed the contrary since strain *C. suis* Rogers 132, a strain with a deletion of both *IScs605* transposases, was the only strain to properly produce recombinants in the absence of counter-selection (Marti et al., 2017). The decreased recombination efficiency of SWA-110 will have to be explored in future studies. For example, a very recent study demonstrated that the ComEC homolog CT339 is crucial for both DNA uptake and lateral gene transfer in *C. trachomatis* (LaBrie et al., 2019), and it is worth exploring whether SWA-110 displays protein alterations in this or other recombination-associated genes.

We analyzed 19 recombinants and determined whether recombinants originating from the same plaque assay have a higher chance of being sibling clones compared to recombinants obtained from another condition of the same co-culture experiment. As expected from our previous study (Marti et al., 2017), while not every pick from the same plaque assay resulted in the acquisition of a sibling clone, there were only seven unique recombinants and 12 recombinants had at least one sibling clone that was identical either upstream and/or downstream of the Tet insertion (Table 4). Interestingly, recombinant 251.1 from Plaque assay group 4 was identical to 256.1 and 259.1 of Plaque assay group 5, indicating that recombination either happened before the monolayers were infected or cross-contamination of the wells occurred. Considering the obligate intracellular nature of *Chlamydia* where replication (Elwell et al., 2016), and therefore recombination, only occurs inside of the cell, and that these recombinants all originate from the same co-culture experiment cross-contamination is the most likely explanation.

We also looked at the average size of Tet-inserts and non-Tet regular inserts. The Tet-insert size range of 20–200 kbp is in line with our preliminary study where two distinct Tet-inserts were described with 55.3 and 175 kbp in length (Marti et al., 2017). Notably, this variation is also comparable to the 98 kbp Tet-insert found in *C. muridarum* post co-culture with the TetR *C. suis* strain R19 (Suchland et al., 2009). In contrast, in intraspecies recombinant studies with *C. trachomatis* (DeMars and Weinfurter, 2008; Jeffrey et al., 2013), fragments of 200–400 kbp were observed regularly.

As a final step, we looked at the precise regions of recombination of the Tet-inserts, but found no specific patterns beyond the fact that *cadA* and *ftsK* were often involved upstream and *nqrF* downstream, which may be explained by their relative distance to the Tet-island. The lack of a specific recombination pattern has already been observed in previous co-culture studies with *C. trachomatis* (Jeffrey et al., 2013) and is also indicative for the high genomic plasticity of *C. suis* previously described in whole-genome studies (Joseph et al., 2016; Seth-Smith et al., 2017). However, considering that only seven samples were truly independent and no statistical analysis could be performed, more whole genomes must be analyzed to identify specific patterns of recombination.

In summary, we developed a co-culture protocol that advanced on previous protocols (Suchland et al., 2009; Marti et al., 2017) to identify factors crucial for the generation of double-resistant recombinants in general and the transfer of the *C. suis* Tet-island in particular. For this purpose, we used three different recent clinical *C. suis* TetR donor strains and co-cultured them with three TetS recipient strains, all of which had been isolated and characterized in previous studies (Wanninger et al., 2016; Seth-Smith et al., 2017). The counter-selection consisted of rifampicin resistance in the TetS strains (RifR). The genomes of TetS strains before and after acquiring rifampicin resistance were examined to further understand the genetic basis of rifampicin/rifamycin resistance in TetS strains. We found that *rpoB* mutations are still the most likely candidates to induce resistance as identified in previous studies (Kutlin et al., 2005; Suchland et al., 2005) but additional analyses of *rpoB* are needed. Using two alternative co-culture protocols and subinhibitory concentrations of tetracycline demonstrated that a co-culture protocol with simultaneous selection increases the recombination efficiency compared to sequential selection and resulted in an allover recombination efficiency of 28.0%. We found that tetracycline did not promote the acquisition of the Tet-island in this study and that co-culture experiments with the TetR strain SWA-110 leads to decreased recombination efficiencies. Finally, we show that sequencing cultures from the same plaque assay produced sibling clones as well as unique recombinants, indicating that the *C. suis* genome is relatively plastic and permissive to lateral gene transfer.

## Data Availability Statement

The datasets presented in this study can be found in online repositories. The names of the repository/repositories and accession number(s) can be found in the article/Supplementary Material.

## Author Contributions

HM, NB, and DD substantially contributed to the conception and design of the work. HM, SB, TR, DD, and NB acquired, analyzed, and interpreted the data. All authors drafted and/or critically revised the manuscript, finally approved the version to be published, and agreed to be accountable for all aspects of the work.

## Conflict of Interest

The authors declare that the research was conducted in the absence of any commercial or financial relationships that could be construed as a potential conflict of interest.
