# Supplementary material for: Generation of Tetracycline and Rifamycin Resistant Chlamydia Suis Recombinants
Source: Front Microbiol. 2021 Jun 30;12:630293. doi: 10.3389/fmicb.2021.630293 (PMC8278220; doi:10.3389/fmicb.2021.630293)
Supplement: Supplementary file 1 [file Data_Sheet_1.zip › MartiH_1_SupplementaryData-1-4.pdf]

Methods

Generation of tetracycline and rifamycin resistant *Chlamydia suis* recombinants

Hanna Marti[1], Sankhya Bommana[2], Timothy D. Read[3,4], Theresa Pesch[1], Barbara Prähauser[1], Deborah Dean[2, 5-7], Nicole Borel[1]

[1]Institute of Veterinary Pathology, Vetsuisse Faculty, University of Zurich, Zurich, Switzerland

[2]Center for Immunobiology and Vaccine Development, UCSF Benioff Children's Hospital Oakland Research Institute, Oakland, CA, United States

[3]Division of Infectious Diseases, Department of Medicine, Emory University School of Medicine, Atlanta, GA, USA

[4]Department of Human Genetics, Emory University School of Medicine, Atlanta, GA, USA

[5]Joint Graduate Program in Bioengineering, University of California, San Francisco, San Francisco, CA, United States

[6]Joint Graduate Program in Bioengineering, University of California, Berkeley, Berkeley, CA, United States

[7]School of Medicine, University of California, San Francisco, San Francisco, CA, United States

Supplementary Data

Supplementary Data 1: Immunofluorescence assay (IFA) and titration by sub-passage

For visualization, immunofluorescence assays (IFA) were performed as described (Leonard et al., 2015). Specifically, cultures were grown on coverslips and fixed in chilled methanol for 10 min prior to replacement with phosphate-buffered saline (PBS, Gibco). Following incubation in blocking solution (1% bovine serum albumin [BSA, Sigma-Aldrich] in PBS) for 30 min, chlamydial inclusions were labeled for 1 h with a 1:200 diluted, monoclonal mouse-antibody that is specific for the *Chlamydiaceae* lipopolysaccharide (LPS, Clone ACI-P; Progen, Heidelberg, Germany). After washing the coverslips three times with PBS, labeled inclusions were visualized with a secondary, 1:500 diluted Alexa Fluor 488-conjugated secondary goat anti-mouse antibody (Molecular Probes, Eugene, OR, USA). Host and chlamydial DNA were stained using 1 µg/ml 4’, 6-diamidino-2’-phenylindole dihydrochloride (DAPI, Molecular Probes). Secondary antibody and DAPI were added to monolayers for 45 min at room temperature prior to washing steps. Finally, coverslips were mounted with FluoreGuard mounting medium (Hard Set; ScyTek Laboratories Inc., Logan, UT, USA) on glass slides and evaluated using a Leica DMLB fluorescence microscope (Leica Microsystems, Wetzlar, Germany) under oil immersion at 1000x magnification with a 100x objective (PL FLUOTAR 100x/1.30, OIL, ‘/0.17/D, Leica Microsystems) and a 10x ocular objective (Leica L-Plan 10x/ 25 M, Leica Microsystems).

Titration by sub-passage was used to determine the infectivity of a chlamydial stock or sample. Briefly, 10 µl of SPG stock or culture was inoculated in duplicate into the first wells and then serially diluted three-fold onto fresh monolayers with glass coverslips. After an incubation period of 48 hours, monolayers were fixed and processed for IFA. The infectivity of the cultures was expressed as inclusion forming units per ml (IFU/ml) and determined by counting 30 ocular grids (area: 0.2775 mm^2) using the 20x objective (PL FLUOTAR 20x/0.50 PH 2, ‘/0.17/B) of the Leica DMLB fluorescence microscope (Leonard et al., 2015).

Supplementary Data 2: Plaque Assay protocol

Following co-culture with a TetR and a TetS *C. suis*, the four wells representing the conditions C1S1, C1S2, C2S1 and C2S2 were passaged twice prior to performing the plaque assay according to previously described protocols (Marti et al., 2017) with minor modifications. In detail, each culture was inoculated separately onto the first well of a 6-well plate containing 2 ml of infection medium per well and then diluted 10-fold resulting in a dilution series from 1:1 to 1:10^5 per plate, which were then centrifuged for 1 h at 1000 g and 37 °C. Following centrifugation, inocula were replaced with infection medium with (Selection 2: C1S2, C2S2) and without (Selection 1: C1S1, C2S1) selective antibiotics and incubated at regular culture conditions for 24 h. Next, 11% SeaKem ME agarose (Lonza; Cat. No. 50011) dissolved in water and mixed with equal volumes of cycloheximide-free infection medium was added to the monolayers (2 ml/well) and topped with another 2 ml of infection medium after the agarose solidified. Following 12-24 h of incubation, the dilution containing only few visible inclusions was selected, of which twelve were picked with a blunt-ended, sterile transfer pipette (Sigma-Aldrich; Cat. No. Z135070-500EA) and used to inoculate 2 ml tubes containing 0.5-1 ml of infection medium. Inocula were mixed thoroughly with a pipet and transferred to fresh monolayers of a 24-well plate, where the resulting 48 picks were inoculated onto fresh cells and passaged every 48 to 72 h for up to six times in the absence of selective antibiotics. Before every passage, monolayers were checked for infection using phase contrast microscopy (Ti Eclipse microscope; Nikon, Tokyo, Japan). Cultures were collected once an infection rate of 50-100% was reached, or if by Passage 6, there were visible inclusions. Picks that did not yield any inclusions by then were considered unsuccessful. Successful picks were further processed to identify recombinants.

Supplementary Data 3: Identification of recombinants by PCR methods

PCR methods were based on a *tetA*(C) PCR detecting the Tet-island, a donor- and a recipient strain-specific PCR.

tetA(C) PCR

The previously published primer pair CS43 (5'-AGCACTGTCCGACCGCTTTG-3') and CS47 (5'-TCCTCGCCGAAAATGACCC-3') targets a 525 bp fragment of the *tetA*(C) gene (Dugan et al., 2004). Cycling conditions consisted of initial denaturation (5 min, 95°C) followed by 35 cycles of denaturation (95°C, 30 s), annealing (59°C, 30 s) and extension (72°C, 1 min) and a final elongation step (72°C) for 10 min. Primer concentration was 0.3 µM per primer, which was mixed with 3 µl DNA template, 1X AmpliTaq Gold™ 360 Master Mix (Thermo Scientific) and water for a final volume of 20 µl. Alternatively, the same primer concentration was used for a mastermix consisting of 1X FastStart PCR Buffer with 20 mM $MgCl_2$ (Roche, Basel, Switzerland) supplemented with 0.5 mM $MgCl_2$ (Roche), 0.2 mM dNTP (Roche), 0.5 U FastStart Taq Polymerase (Roche) with a final volume of 20 µl.

Strain-specific PCR

Table S1A lists the primers used for each strain. Recipient strains were generally tested with the primer pair SWA-TS_1F/SWA-TS_1R. Mastermix composition was identical to that of the *tet*A(C) PCR. Cycling conditions comprised of a denaturation step for 5 min followed by 35 cycles of 95 °C for 1 min, an annealing step for 1 min, an elongation step at 72 °C for 1.5 min, and a final extension for 10 min (72 °C). The annealing temperature was 65 °C except for the SWA-107 primer pair (62 °C) and the S45 RIF primer pair (54 °C).

Interpretation and subsequent characterization of the PCR results is listed in Table S1B.

Table S1A: List of strain-specific primers

Strain	Primer name	Primer sequence (5'-3')	Gene	Locus tag[1]	Size (bp)
SWA-107	107_2F	GCAGGAATGTTTCGAATTG	*pld 6* (PZ)	Suis_9_00675	280
	107_2R	AGAGATCAAGCGTACCAC			
SWA-110	110_3F	CTCCTTCAAATGCAGAAACCGTAACA	*pmpB*	Suis_9_00954	656
	110_3R	CGATTCAGATTGGGAAGATGCTGT			
SWA-141	SWA-141_1F	GGAACCTCATCCACAACAG	*pmpB*	Suis_9_00954	608
	SWA 141_2F	TGTGGGGACGTTGGTGA			
Recipient strains	SWA-TS_1F	CCAGTTCGGATCATAGACCT	*pmpB*	Suis_9_00954	400
	SWA-TS_1R	GATGTTTGGGAGTTTGGTAG			
S45/6 RIF	S45_1F	CCACAAACTCTCAACAG	Intergenic *pmpBC*	-	1439
	S45_1R	TCTCCAGATTCAGTTGG			
94 Ry	94_2F	TGCCATAGGAACCTTGGGA	*pmpB*	Suis_9_00954	963
	94_2R	CTTGCTCCGCCGCTATTACT			
111 Ry	111_1F	GGAAGCTCCAAGAAAGATACCAT	*pmpB*	Suis_9_00954	560
	111_1R	CAGCAGGGTTGTCTGGAGT			

[1]The locus tag is derived from the annotated genome of S45 (Joseph et al., 2016).

Table S1B: Categorization of successful plaque assay picks according to PCR results

Category	*tetA*(C) PCR	Donor PCR	Recipient PCR
Putative recombinant	Pos	Neg	Pos
Mixed infection	Pos	Pos	Pos
Tet-island negative culture	Neg	Neg	Pos
Other culture	Other combinations; not listed above		

Supplementary Data 4: Stability assay

Stability assays were modified from a previously established protocol (Marti et al., 2017): Following titer determination, fresh monolayers containing infection medium were inoculated with a multiplicity of infection (MOI) of 0.1 of the putative recombinant of interest, centrifuged for 1 h at 1000 g and 25 °C and incubated for five passages (final volume: 1 ml). Next, cultures were either treated with selective antibiotics before they were cultured again until Passage 10 in the absence of antibiotics, or they were subjected directly to PCR analysis and antibiotic susceptibility assay (Figure S1).

PCR analysis was performed as described above. For antibiotic susceptibility testing, 100-500 µl of the Passage 5 and the Passage 10 culture was added to 3 ml of infection medium and evenly distributed onto 24 wells of a 96-well plate (100 µl/well), centrifuged (1000 g, 1h, 25 °C) and one row (12 wells) was replaced with either fresh infection medium (drug-free control) or infection medium containing tetracycline (0.015 to 16 µg/ml, 11 wells, two-fold dilution between wells). The second row either contained rifamycin (same as tetracycline) or rifampicin (2.4E-4 to 0.25 µg/ml) depending on the recipient strain (S45 RIF: rifampicin; 94 Ry and 111 Ry: rifamycin). After 48 hours of incubation, 96-well plates were fixed in methanol, processed for IFA and the approximate MIC was determined using the Ti Eclipse microscope and MIC criteria developed previously (Marti et al., 2018), specifically the determination of the 'initial or approximate phenotype'. Briefly, MIC was determined as two times the concentration where the majority of inclusions were changed in size and morphology and the concentration where the number of inclusions was strongly reduced. This approximate phenotype was sufficient to determine whether the cultures were resistant or sensitive to the antibiotic of choice.

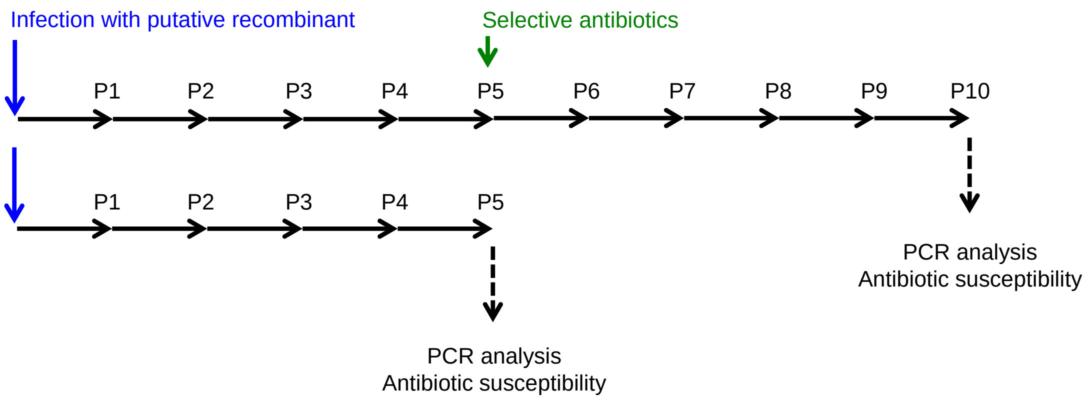

Figure S1: Experimental design of the stability assay. Shown is the experimental design of the stability assay. Following infection with the putative recombinant (blue), cultures are grown for five to ten passages (P) in the absence of selective antibiotics prior to PCR analysis

and testing the antibiotic susceptibility of the cultures to tetracycline and rifamycin/rifampicin.
