# Supplementary material for: Generation of Tetracycline and Rifamycin Resistant Chlamydia Suis Recombinants
Source: Front Microbiol. 2021 Jun 30;12:630293. doi: 10.3389/fmicb.2021.630293 (PMC8278220; doi:10.3389/fmicb.2021.630293)
Supplement: Supplementary file 1 [file Data_Sheet_1.zip › MartiH_1_SupplementaryData-8.pdf]

Methods

Generation of tetracycline and rifamycin resistant *Chlamydia suis* recombinants

Hanna Marti[1], Sankhya Bommana[2], Timothy D. Read[3,4], Theresa Pesch[1], Barbara Prähauser[1], Deborah Dean[2, 5-7], Nicole Borel[1]

[1]Institute of Veterinary Pathology, Vetsuisse Faculty, University of Zurich, Zurich, Switzerland

[2]Center for Immunobiology and Vaccine Development, UCSF Benioff Children's Hospital Oakland Research Institute, Oakland, CA, United States

[3]Division of Infectious Diseases, Department of Medicine, Emory University School of Medicine, Atlanta, GA, USA

[4]Department of Human Genetics, Emory University School of Medicine, Atlanta, GA, USA

[5]Joint Graduate Program in Bioengineering, University of California, San Francisco, San Francisco, CA, United States

[6]Joint Graduate Program in Bioengineering, University of California, Berkeley, Berkeley, CA, United States

[7]School of Medicine, University of California, San Francisco, San Francisco, CA, United States

Supplementary Data

Supplementary Data 8: Flow diagram of co-culture protocols and factors

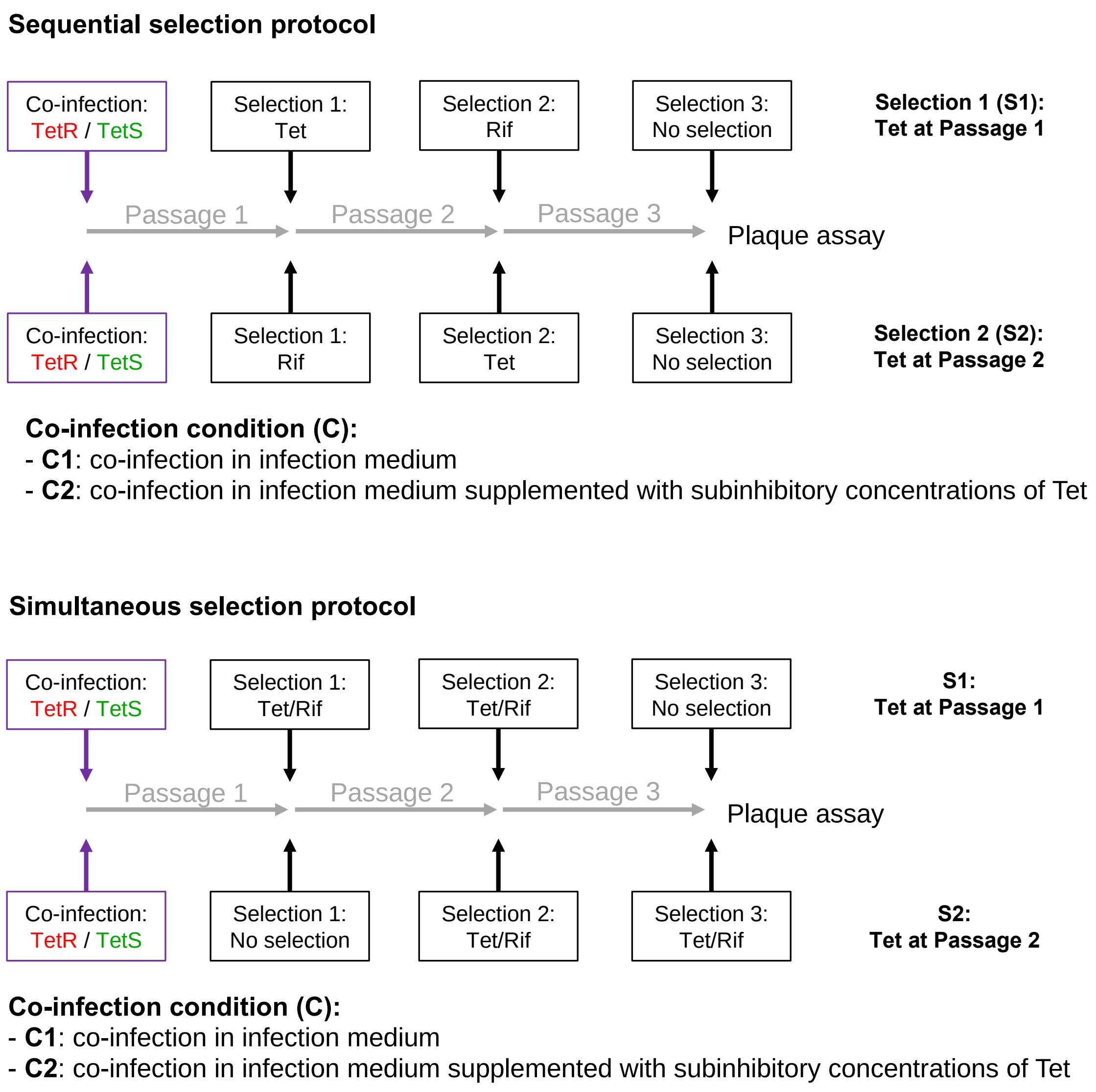

Figure S2: Overview of co-culture protocol. Shown are the co-culture protocols and factors analyzed in this study. The top panel shows the first selection protocol (sequential selection) where selective antibiotics were applied sequentially and the second (simultaneous selection) where the selective antibiotics were applied simultaneously. Furthermore, tetracycline selection was either added at the first (S1) or the second (S2) passage. Finally, co-culture was either performed in regular infection medium (C1) or in the presence of subinhibitory concentration of tetracycline (C2) resulting in four separate conditions: 1) C1S1, 2) C1S2, 3) C2S1 and 4) C2S2.
