# Supplementary material for: Generation of Tetracycline and Rifamycin Resistant Chlamydia Suis Recombinants
Source: Front Microbiol. 2021 Jun 30;12:630293. doi: 10.3389/fmicb.2021.630293 (PMC8278220; doi:10.3389/fmicb.2021.630293)
Supplement: Supplementary file 1 [file Data_Sheet_1.zip › MartiH_1_SupplementaryData-9.pdf]

Methods

# Generation of tetracycline and rifamycin resistant *Chlamydia suis* recombinants

Hanna Marti[1], Sankhya Bommana[2], Timothy D. Read[3,4], Theresa Pesch[1], Barbara Prähauser[1], Deborah Dean[2, 5-7], Nicole Borel[1]

[1]Institute of Veterinary Pathology, Vetsuisse Faculty, University of Zurich, Zurich, Switzerland

[2]Center for Immunobiology and Vaccine Development, UCSF Benioff Children's Hospital Oakland Research Institute, Oakland, CA, United States

[3]Division of Infectious Diseases, Department of Medicine, Emory University School of Medicine, Atlanta, GA, USA

[4]Department of Human Genetics, Emory University School of Medicine, Atlanta, GA, USA

[5]Joint Graduate Program in Bioengineering, University of California, San Francisco, San Francisco, CA, United States

[6]Joint Graduate Program in Bioengineering, University of California, Berkeley, Berkeley, CA, United States

[7]School of Medicine, University of California, San Francisco, San Francisco, CA, United States

**Supplementary Data**

*Supplementary Data 9: Comparative p-values*

**Table S5: Overview of comparative p-values as determined by Fisher's exact test***

| **Mating pair** | **1** | **2** | **3** | **4** | **5** | **6** | **7** |
|---|---|---|---|---|---|---|---|
| **1: SWA-141/S45 RIF** | - | 0.8945 | 0.4173 | 0.3155 | 0.042 | <0.0001 | <0.0001 |
| **2: SWA-141/94 Ry** | 0.8945 | - | 0.2942 | 0.2087 | 0.0766 | <0.0001 | <0.0001 |
| **3: SWA-141/111 Ry** | 0.4173 | 0.2942 | - | 0.8247 | 0.0024 | <0.0001 | <0.0001 |
| **4: SWA-107/94 Ry** | 0.3155 | 0.2087 | 0.8247 | - | 0.0017 | <0.0001 | <0.0001 |
| **5: SWA-107/111 Ry** | 0.042 | 0.0766 | 0.0024 | 0.0017 | - | 0.0194 | <0.0001 |
| **6: SWA-110/94 Ry** | <0.0001 | <0.0001 | <0.0001 | <0.0001 | 0.0194 | - | 0.0329 |
| **7: SWA-110/111 Ry** | <0.0001 | <0.0001 | <0.0001 | <0.0001 | <0.0001 | 0.0329 | - |

*The Bonferroni correction (21 comparisons) resulted in a significant p-value ≤ 0.0024.
