# Supplementary material for: Generation of Tetracycline and Rifamycin Resistant Chlamydia Suis Recombinants
Source: Front Microbiol. 2021 Jun 30;12:630293. doi: 10.3389/fmicb.2021.630293 (PMC8278220; doi:10.3389/fmicb.2021.630293)
Supplement: Supplementary file 1 [file Data_Sheet_1.zip › MartiH_1_SupplementaryData-10.pdf]

Methods

Generation of tetracycline and rifamycin resistant *Chlamydia suis* recombinants

Hanna Marti[1], Sankhya Bommana[2], Timothy D. Read[3,4], Theresa Pesch[1], Barbara Prähauser[1], Deborah Dean[2, 5-7], Nicole Borel[1]

[1]Institute of Veterinary Pathology, Vetsuisse Faculty, University of Zurich, Zurich, Switzerland

[2]Center for Immunobiology and Vaccine Development, UCSF Benioff Children's Hospital Oakland Research Institute, Oakland, CA, United States

[3]Division of Infectious Diseases, Department of Medicine, Emory University School of Medicine, Atlanta, GA, USA

[4]Department of Human Genetics, Emory University School of Medicine, Atlanta, GA, USA

[5]Joint Graduate Program in Bioengineering, University of California, San Francisco, San Francisco, CA, United States

[6]Joint Graduate Program in Bioengineering, University of California, Berkeley, Berkeley, CA, United States

[7]School of Medicine, University of California, San Francisco, San Francisco, CA, United States

Supplementary Data

<u>Supplementary Data 10: Recombinant Analysis</u>

Group 1: Experiment no. 4 SWA-107 / 94 Ry (C1S2)

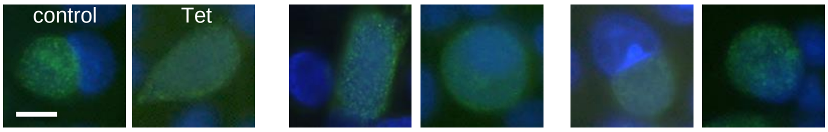

157.1 160.1 161.1

Group 2: Experiment no. 5 SWA-141 / S45 RIF (C1S2)

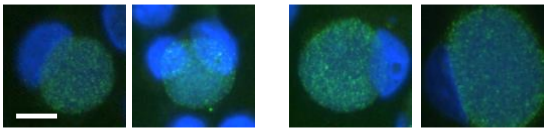

208.1 212.1

Group 3: Experiment no. 5 SWA-141 / S45 RIF (C2S2)

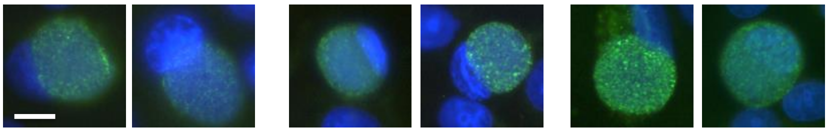

230.1 232.1 236.1

Group 4: Experiment no. 6 SWA-141 / 94 Ry (C1S1)

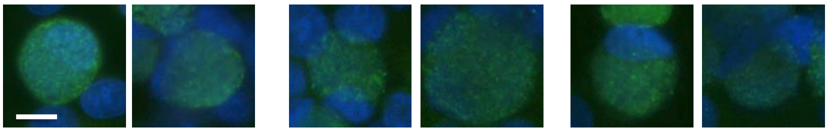

241.1 246.1 251.1

Group 5: Experiment no. 6 SWA-141 / 94 Ry (C1S2)

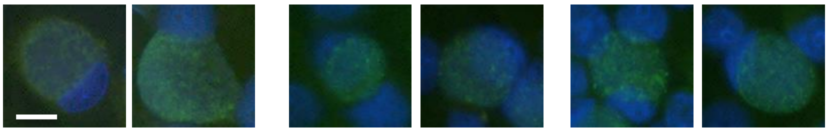

253.1 256.1 259.1

Group 6: Experiment no. 6 SWA-141 / 94 Ry (C2S2)

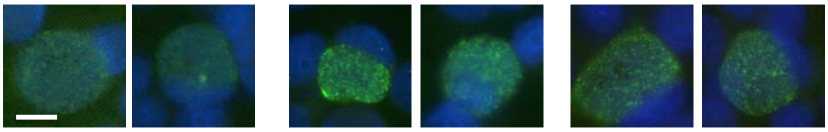

277.1 281.1 287.1

Group 7: Experiment no. 12 SWA-110 / 111 Ry (C2S2)

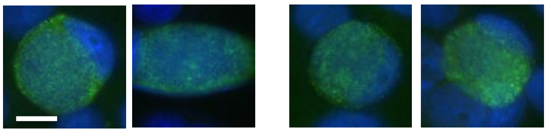

569.1 572.1

Control

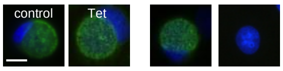

TetR TetS

Figure S3: Tetracycline resistance screen results. Per recombinant, two monolayers were infected with MOI 0.5, centrifuged and replaced without (control, left) or with (Tet, right) 0.5 µg/ml of tetracycline prior to incubation for 48 h, fixation and IFA. Shown are representative images for the nineteen recombinants used for whole-genome sequencing as well as a TetR (SWA-141) and a TetS (S45 RIF) control. The size bar indicates 10 µm.
