# Supplementary material for: Generation of Tetracycline and Rifamycin Resistant Chlamydia Suis Recombinants
Source: Front Microbiol. 2021 Jun 30;12:630293. doi: 10.3389/fmicb.2021.630293 (PMC8278220; doi:10.3389/fmicb.2021.630293)
Supplement: Supplementary file 1 [file Data_Sheet_1.zip › MartiH_1_SupplementaryData-14.pdf]

Supplementary Data 14: RpoB amino acid alignment of *C. suis* S45, S45 RIF and PDB template 5ual. RpoB amino acid alignment was created using Clustal Omega and the residues involved in rifampicin binding (cyan), previously reported mutations that confer resistance to RIF (red) and mutation cluster I-III (I: codons 507-533; II: 563-572, III: 687) (in bold) were annotated based on crystal structures of *Escherichia coli* 5ual available from NCBI, PDB and Goldstein et al., 2014). Amino acid substitution (yellow) in the *C. suis* S45 RIF relative to S45 is also annotated.

```
5ual         MVYSYTEKKRIRKDFGKRPQVLDVPYLLSIQLDSFQKFIEQD----PEGQYGLEAAFRSV    56
S45_RIF      -MFKC----PERVSVKKKEDILDLPNLVEVQIKSYKQFLQIGKLPEERENIGLEEVFREI    55
S45          -MFKC----PERVSVKKKEDILDLPNLVEVQIKSYKQFLQIGKLPEERENIGLEEVFREI    55
              ::.       * .. *: ::**:* *:.:*:.*:::*:: .     . : *** .**.:

5ual         FPIQSYSGNSELQYVSYRLGEPVFDVQECQIRGVTYSAPLRVKLRLVIYEREAPEGTVKD    116
S45_RIF      FPIKSYNEATILEYLSYNLGVPKYSPEECIRRGITYSVTLKVRFRLTD---------ETG    106
S45          FPIKSYNEATILEYLSYNLGVPKYSPEECIRRGITYSVTLKVRFRLTD---------ETG    106
             ***:**.  : *:*:**.** * :. :**  **:***. *:*::**.           ..

5ual         IKEQEVYMGEIPLMTDNGTFVINGTERVIVSQLHRSPGVFFDSDKGKTHSSGKVLYNARI    176
S45_RIF      IKEEEVYMGTIPIMTDKGTFIINGAERVVVSQVHRSPGINFEQE---KHSKGNVLFSFRI    163
S45          IKEEEVYMGTIPIMTDKGTFIINGAERVVVSQVHRSPGINFEQE---KHSKGNVLFSFRI    163
             ***:***** **:***:***:***:***:***:*****: *:.:   .**.*:**:. **

5ual         IPYRGSWLDFEFDPKDNLFVRIDRRR---KLPATIILRALNYTTEQ-ILDLFFEKVIFEI    232
S45_RIF      IPYRGSWLEAVFDINDLIYIHIDRKKRRRKILAMTFIRALGYSTDADIIEEFFAVEERAL    223
S45          IPYRGSWLEAVFDINDLIYIHIDRKKRRRKILAMTFIRALGYSTDADIIEEFFAVEERAL    223
             ********:  ** :* ::::***::   *: *  ::***.*:*:  *:: **      :

5ual         RDNKLQMELVPERLRGETASFDIEANGKVYVEKGRRITARHIRQLEKDDVKLIEVPVEYI    292
S45_RIF      HSEKDF---------------------------------------------------VSL    232
S45          HSEKDF---------------------------------------------------VSL    232
             :.:*                                                       :

5ual         AGKVVAKDYIDESTGELICAANMELSLDLLAKLSQSGHKRIETLFTNDLDHGPYISETLR    352
S45_RIF      VGKVLADNVVDADSSLVYGKAGEKLSTAMLKRILDAGVQSLKVAVGA--DENHPIIKMLA    290
S45          VGKVLADNVVDADSSLVYGKAGEKLSTAMLKRILDAGVQSLKVAVGA--DENHPIIKMLA    290
             .***:*.: :* .:. :   *. :**  :* :: ::* : ::. .    *..  * : *

5ual         VDPTNDRLSALVEIYRMMRPGEPPTREAAESLFENLFFSEDRYDLSAVGRMKFNRSLL--    410
S45_RIF      KDPTDSYEAALKDFYRRLRPGEPATLANARSTIMRLFFDAKRYNLGRVGRYKLNKKLGFP    350
S45          KDPTDSYEAALKDFYRRLRPGEPATLANARSTIMRLFFDAKRYNLGRVGRYKLNKKLGFP    350
              ***:.  :** ::** :***** *   *.* : .***. .**:*. *** *:*:.*

5ual         -REEIEGSGILSKDDIIDVMKKLIDIRNG--KGEVDDIDHLGNRRIRSVGEMAENQFRVG    467
S45_RIF      LDDETLSQVTLRKEDVIGALKYLIRLRMGDEKTSIDDIDHLANRRVRSVGELIQNHCRSG    410
S45          LDDETLSQVTLRKEDVIGALKYLIRLRMGDEKTSIDDIDHLANRRVRSVGELIQNHCRSG    410
               :*  ..  * *:*:*..:* ** :*  *  * .:******.***:*****: :*: * *

5ual         LVRVERAVKERLSLGDL--DTLMPQDMINAKPISAAVKEFFGSSQLSQFMDQNNPLSEIT    525
S45_RIF      LARMEKIVRERMNLFDFSSDTLTPGKIISAKGLVSVLKDFFSRSQLSQFMDQTNPVAELT    470
S45          LARMEKIVRERMNLFDFSSDTLTPGKIISAKGLVSVLKDFFSRSQLSQFMDQTNPVAELT    470
             *.*:*: *:**:.* *:  *** * .:*.** : :.:*:**. *********.**::*:*

5ual         HKRRILALGPGGLTRERAGFEVRDVHPTHYGRVCPIETPEGPNIGLINSLSVYAQTNEYG    585
S45_RIF      HKRRLSALGPGGLNRERAGFEVRDVHASHYGRICPIETPEGPNIGLITSLSSFAKINEFE    530
S45          HKRRLSALGPGGLNRERAGFEVRDVHASHYGRICPIETPEGPNIGLITSLSSFAKINEFG    530
             ****: *******.************ :****:**************.*** :*: **:

5ual         FLETPYRKVTDGVVTDEIHYLSAIEEGNYVIAQANSNLDEEGHFVEDLVTCRSKGESSLF    645
S45_RIF      FIETPYRVVRDGIVTDEIEYMTADVEEDCVIAQASAELDEYNMFKNPVCWARYKGEAFEA    590
S45          FIETPYRVVRDGIVTDEIEYMTADVEEDCVIAQASAELDEYNMFKNPVCWARYKGEAFEA    590
             *:***** * **:*****.*::*  * : *****.::*** . * : :  .* ***:
```

```
5ual         SRDQVDYMDVSTQQVVSVGASLIPFLEHDDANRALMGANMQRQAVPTLRADKPLVGTGME    705
S45_RIF      DTSTVTHMDVSPKQLVSVVTGLIPFLEHDDANRALMGSNMQRQAVPLLKTEAAIVGTGLE    650
S45          DTSTVTHMDVSPKQLVSVVTGLIPFLEHDDANRALMGSNMQRQAVPLLKTEAAIVGTGLE    650
             . . * :**** :*:*** :.****************:******** *:::  :****:*

5ual         RAVAVDSGVTAVAKRGGVVQYVDASRIVIKVNEDEMYPGEAGIDIYNLTKYTRSNQNTCI    765
S45_RIF      GRAAKDSGAIIVAQEDGIVEYVDSYEIVVAKKNNP-----TLKDKYQLKKFLRSNSGTCI    705
S45          GRAAKDSGAIIVAQEDGIVEYVDSYEIVVAKKNNP-----TLKDKYQLKKFLRSNSGTCI    705
               .* ***.  **:..*:*:***: .**:  :::      :  * *:*.*: ***..***

5ual         NQMPCVSLGEPVERGDVLADGPSTDLGELALGQNMRVAFMPWNGYNFEDSILVSERVVQE    825
S45_RIF      NQTPLCSVGDVVTHGDVLADGPATDKGELALGKNVLVAFMPWYGYNFEDAIIISEKLIKQ    765
S45          NQTPLCSVGDVVTHGDVLADGPATDKGELALGKNVLVAFMPWYGYNFEDAIIISEKLIKQ    765
             ** *  *:*: * :********:** ******:*: ****** ******:*::**:::::

5ual         DRFTTIHIQELACVSRDTKLGPEEITADIPNVGEAALSKLDESGIVYIGAEVTGGDILVG    885
S45_RIF      DAYTSIYIEEFELTARDTKLGKEEITRDIPNVSEEVLANLGEDGIVRIGAEVKPGDILVG    825
S45          DAYTSIYIEEFELTARDTKLGKEEITRDIPNVSEEVLANLGEDGIVRIGAEVKPGDILVG    825
             * :*:*:*:*:   .:****** **** *****.* .*::*.*.*** *****. ******

5ual         KVTPKGETQLTPEEKLLRAIFGEKASDVKDSSLRVPNGVSGTVIDVQVFTRDGVEKD-KR    944
S45_RIF      KITPKSETELAPEERLLRAIFGEKAADVKDASLTVPPGTEGVVMDVKVFSRKDRLSKSDD    885
S45          KITPKSETELAPEERLLRAIFGEKAADVKDASLTVPPGTEGVVMDVKVFSRKDRLSKSDD    885
             *:***.**:*:***:*********:****:** ** *..*.*:**:**:*..  .. .

5ual         ALEIEEMQLKQAKKDLSEELQILEAGLFSRIRAVL------------------VAGG--V    984
S45_RIF      ELVEEAVHLKDLQKEYKAQLAQLKVEHREKLGALLLNEKAPAAIIHRRSADILVQEGAVF    945
S45          ELVEEAVHLKDLQKEYKAQLAQLKVEHREKLGALLLNEKAPAAIIHRRSADILVQEGAVF    945
              *  * ::**: :*: . :*  *:.   .:: *:*                  *  *  .

5ual         EAEKLDKLPRDRWLELGLTDEEKQNQLEQLAEQYDELKHEFEKKLEAKRRKITQ-GDDLA    1043
S45_RIF      DQETIELLEREPLVDLLMAPCDMYDVLKEILSSYEIAVQRLEVNYKTEAEHIKEGDADLD    1005
S45          DQETIELLEREPLVDLLMAPCDMYDVLKEILSSYEIAVQRLEVNYKTEAEHIKEGDADLD    1005
             : *.:: * *:  ::* ::  :  : *::: ..*:   :.:* : ::: .:*.: . **

5ual         PGVLKIVKVYLAVKRRIQPGDKMAGRHGNKGVISKINPIEDMPYDENGTPVDIVLNPLGV    1103
S45_RIF      HGVIRQVKVYVASKRKLQVGDKMAGRHGNKGVVSKIVPEADMPFLANGETVQMILNPLGV    1065
S45          HGVIRQVKVYVASKRKLQVGDKMAGRHGNKGVVSKIVPEADMPFLANGETVQMILNPLGV    1065
              **:: ****:* **::* *************:*** *  ***:  **  *:::******

5ual         PSRMNIGQILETHLGMAAKGIGDKINAMLKQQQEVAKLREFIQRAYDLGADVRQKVDLST    1163
S45_RIF      PSRMNLGQVLETHLGYAAKTAG--------------------------------------    1087
S45          PSRMNLGQVLETHLGYAAKTAG--------------------------------------    1087
             *****:**:****** ***  *

5ual         FSDEEVMRLAENLRKGMPIATPVFDGAKEAEIKELLKLGDLPTSGQIRLYDGRTGEQFER    1223
S45_RIF      ----------------IYVKTPVFEGFPESRIWDMMIEQGLPEDGKSYLFDGKTGERFDS    1131
S45          ----------------IYVKTPVFEGFPESRIWDMMIEQGLPEDGKSYLFDGKTGERFDS    1131
                             : : ****:*  *:.* :::   .** .*:  *:**:***:*:

5ual         PVTVGYMYMLKLNHLVDDKMHARSTGSYSLVTQQPLGGKAQFGGQRFGEMEVWALEAYGA    1283
S45_RIF      KVVVGYIYMLKLSHLIADKIHARSIGPYSLVTQQPLGGKAQMGGQRFGEMEVWALEAYGV    1191
S45          KVVVGYIYMLKLSHLIADKIHARSIGPYSLVTQQPLGGKAQMGGQRFGEMEVWALEAYGV    1191
              *.***:*****.**: **:**** * **************:*****************.

5ual         AYTLQEMLTVKSDDVNGRTKMYKNIVDGNHQMEPGMPESFNVLLKEIRSLGINIELEDE-    1342
S45_RIF      AHMLQEILTVKSDDVSGRTRIYESIVKGENLLRSGTPESFNVLIKEMQGLGLDVRPMVVD    1251
S45          AHMLQEILTVKSDDVSGRTRIYESIVKGENLLRSGTPESFNVLIKEMQGLGLDVRPMVVD    1251
             *: ***:********.***::*:.**.*:: :. * *******:**::.**:::.

5ual         -1342
S45_RIF      A1252
S45          A1252
```
